# Inhibition of MAGL activates the Keap1/Nrf2 pathway to attenuate glucocorticoid‐induced osteonecrosis of the femoral head

**DOI:** 10.1002/ctm2.447

**Published:** 2021-06-01

**Authors:** Ning Yang, Houyi Sun, Yi Xue, Weicheng Zhang, Hongzhi Wang, Huaqiang Tao, Xiaolong Liang, Meng Li, Yaozeng Xu, Liang Chen, Liang Zhang, Lixin Huang, Dechun Geng

**Affiliations:** ^1^ Department of Orthopaedics The First Affiliated Hospital of Soochow University Soochow University Suzhou China; ^2^ Department of Orthopaedics Changshu Hospital Affiliated to Nanjing University of Traditional Chinese Medicine Changshu China; ^3^ Department of Orthopaedics, Beijing Friendship Hospital Capital Medical University Beijing China

**Keywords:** apoptosis, glucocorticoids, monoacylglycerol lipase, osteonecrosis of the femoral head, oxidative stress

## Abstract

Glucocorticoids (GCs) are used in treating viral infections, acute spinal cord injury, autoimmune diseases, and shock. Several patients develop GC‐induced osteonecrosis of the femoral head (ONFH). However, the pathogenic mechanisms underlying GC‐induced ONFH remain poorly understood. GC‐directed bone marrow mesenchymal stem cells (BMSCs) fate is an important factor that determines GC‐induced ONFH. At high concentrations, GCs induce BMSC apoptosis by promoting oxidative stress. In the present study, we aimed to elucidate the molecular mechanisms that relieve GC‐induced oxidative stress in BMSCs, which would be vital for treating ONFH. The endocannabinoid system regulates oxidative stress in multiple organs. Here, we found that monoacylglycerol lipase (MAGL), a key molecule in the endocannabinoid system, was significantly upregulated during GC treatment in osteoblasts both in vitro and in vivo. MAGL expression was positively correlated with expression of the NADPH oxidase family and apoptosis‐related proteins. Functional analysis showed that MAGL inhibition markedly reduced oxidative stress and partially rescued BMSC apoptosis. Additionally, in vivo studies indicated that MAGL inhibition effectively attenuated GC‐induced ONFH. Pathway analysis showed that MAGL inhibition regulated oxidative stress in BMSCs via the Kelch‐like ECH‐associated protein 1 (Keap1)/nuclear factor erythroid 2‐related factor 2 (Nrf2) pathway. The expression of Nrf2, a major regulator of intracellular antioxidants, was upregulated by inhibiting MAGL. Nrf2 activation can mimic the effect of MAGL inhibition and significantly reduce GC‐induced oxidative damage in BMSCs. The beneficial effects of MAGL inhibition were attenuated after the blockade of the Keap1/Nrf2 antioxidant signaling pathway. Notably, pharmacological blockade of MAGL conferred femoral head protection in GC‐induced ONFH, even after oxidative stress responses were initiated. Therefore, MAGL may represent a novel target for the prevention and treatment of GC‐induced ONFH.

## Graphical abstract

Schematic illustration of MAGL inhibition‐mediated protection from GC‐induced oxidative stress damage. MAGL inhibition suppresses GC‐induced NADPH oxidative isozymes upregulation through activation of Keap1/Nrf2 pathway. Reduced intracellular ROS production results in a blockade of the mitochondrial apoptosis pathway.

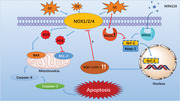

## 1 INTRODUCTION

Glucocorticoids (GCs) are used extensively for the treatment of viral infections, acute spinal cord injury, autoimmune diseases, shock, and other diseases, owing to their excellent anti‐inflammatory and immunosuppressive effects. Several patients suffer from GC‐induced osteonecrosis of the femoral head (ONFH), and joint replacement is usually the final recourse.^1^

The pathogenic mechanisms underlying GC‐induced ONFH remain poorly understood. However, the reduced number of osteoblasts is one of the primary characteristic features of ONFH.^2^ Some studies have shown that excessive use of GCs leads to bone marrow‐derived mesenchymal stem cell (BMSC) apoptosis.^3^, ^4^, ^5^, ^6^ GCs can induce endoplasmic reticulum stress in BMSCs and negatively regulate B‐cell lymphoma 2 (BCL‐2) expression.^7^ Consequently, the BCL‐2/BCL‐2‐associated X protein (BAX) complex dissociates, and BAX content in the mitochondria increases. Activation of Bax will induce the discharge of cytochrome C.^8^, ^9^ Next, the caspase 9/caspase 3 signaling pathway is initiated, which triggers cell apoptosis.^10^, ^11^, ^12^, ^13^ Therefore, suppressing BMSC apoptosis may help to prevent GC‐induced ONFH in its early stages.

Recently, investigators have shown that a high dose of GCs can cause oxidative stress in osteoblasts.^14^ Under severe oxidative stress, the intracellular accumulation of excess reactive oxygen species (ROS) leads to increased endoplasmic reticulum stress, mitochondrial DNA (mtDNA) damage, mitochondrial dysfunction, and eventually activation of the BCL‐2/BAX apoptosis pathway.^15^ These reports provide useful insights for preventing GC‐induced apoptosis in BMSCs. Therefore, reduced ROS generation in BMSCs may exert a positive therapeutic effect against GC‐induced ONFH.

In the past decade, monoacylglycerol lipase (MAGL), the primary hydrolyzing enzyme of 2‐arachidonoylglycerol (2AG), has attracted widespread attention. MAGL acts as a key mediator in cells and regulates a diverse range of biological processes, such as lipid metabolism, pain, inflammation, and tumor invasion.^16^, ^17^, ^18^, ^19^ MAGL inhibition can alleviate oxidative stress by elevating 2AG levels during the treatment of hepatic injury and traumatic brain injury.^20^, ^21^ However, it is unclear whether MAGL regulation has a therapeutic value in the treatment of GC‐induced ONFH, and the mechanism through which it occurs remains unknown.

In this study, we induced apoptosis in BMSCs using methylprednisolone sodium succinate (MP) to assess whether MAGL inhibition can provide early protection against GC‐induced ONFH by alleviating oxidative stress levels and to investigate the signaling pathways involved. Our experimental results clearly showed that MAGL inhibition effectively improved GC‐induced ONFH by relieving oxidative damage. Notably, our results showed that the protective effects of MAGL inhibition are mediated via the Kelch‐like ECH‐associated protein 1 (Keap1)/nuclear factor erythroid 2‐related factor 2 (Nrf2) antioxidant signaling pathway. Our findings indicate that MAGL inhibition may represent a novel strategy for the prevention and treatment of GC‐induced ONFH.

## 2 MATERIALS AND METHODS

### 2.1 Chemicals and antibodies

Detailed information on the chemicals, primers, antibodies, lentiviral vectors, siRNAs, and plasmids are outlined in Table S1.

### 2.2 Animal studies

Sprague–Dawley (SD) rats (male, age: 10 weeks, weight: 400 ± 50 g) were obtained from the Laboratory Animal Center of Soochow University. The GC‐induced ONFH model was established as follows: Lipopolysaccharide (LPS, 40 μg/kg) was intraperitoneally injected once daily from day 1 to 3, and MPSS (60 mg/kg) was intramuscularly injected once daily for the following four consecutive days. Thirty‐two SD rats were randomized into four groups (*n* = 8): (1) DMSO only (control group); (2) MP and LPS (model group); (3) model group rats treated with MJN110 (10 mg/kg per day, i.p. injection), where MJN110 was administered 1 h before the first LPS injection (pretreatment group); and (4) model group rats treated with MJN110 (10 mg/kg per day, i.p. injection), where MJN110 was administered 3 h after the last MP injection (posttreatment group). The MJN110 dose used was based on that reported in previous studies.^22^, ^23^, ^24^ The femoral head and long bone samples were harvested at 6 weeks after the establishment of the model. The Ethics Committee of the First Affiliated Hospital of Soochow University approved all animal experiments.

### 2.3 Micro‐computed tomography scans

The femoral heads of rats were scanned and analyzed using high‐resolution micro‐computed tomography (micro‐CT) SkyScan 1176 (Bruker, Aartselaar, Belgium). A pair of specimens was placed in a micro‐CT test tube cup. The scanning parameters were 70 kV, 141 mA, and 1750 ms, with a spatial resolution of 18 μm. The following parameters were analyzed using the CT Analyzer software (Bruker): bone volume (BV, mm^3^), bone volume fraction (BV/TV, %), trabecular thickness (Tb.Th, mm), and trabecular spacing (Tb.Sp, mm).

### 2.4 Histological and immunohistochemical analysis

The femoral head samples were harvested at 6 weeks after the establishment of the model. After 48 h of fixation and 4 weeks of decalcification, the femoral head samples were embedded in paraffin and sectioned. The protein expression of MAGL, NOX1, NOX4, and Nrf2 was evaluated via immunohistochemical analysis (all antibodies were obtained from Abcam, Shanghai, China). The sections were conventionally dewaxed, rehydrated, and subjected to antigen retrieval, followed by blocking with horse serum for 30 min. Next, primary antibodies and the corresponding secondary antibodies were added dropwise to the specimens, and the signal was developed using 3,3ʹ‐diaminobenzidine. Finally, the sections were counterstained with hematoxylin, dehydrated, made transparent, and mounted using neutral resins.

**Highlights**

1. The expression of monoacylglycerol lipase (MAGL) in BMSCs was enhanced on glucocorticoids (GC) stimulation.
2. The expression of MAGL positively correlated with the expression of NADPH oxidase and apoptosis‐related proteins.
3. MAGL inhibition regulated oxidative stress in BMSCs via the Kelch‐like ECH‐associated protein 1 (Keap1)/Nuclear factor erythroid 2‐related factor 2 (Nrf2) pathway.
4. Pharmacological blockade of MAGL could confer significant femoral head protection even when administered after initiation of GC‐induced oxidative stress.

### 2.5 Hematoxylin and eosin staining

The femoral heads of rats were immersed in 4% paraformaldehyde for 48 h. After 4 weeks of decalcification in 10% ethylenediaminetetraacetic acid, the specimens were dehydrated, paraffin embedded, sliced (6 μm), and mounted onto glass slides. After hematoxylin and eosin (H&E) staining, the sections were mounted with neutral resins and observed under an AxioCam HRC microscope (Carl Zeiss, Oberkochen, Germany).

### 2.6 Cell culture

BMSCs were collected from SD rats (6‐week old), as previously described.^25^ BMSCs were cultured in alpha modified Eagle's medium (α‐MEM; Caygen, Soochow, China) supplemented with 10% fetal bovine serum (Gibco) and in a humidified incubator with 5% CO_2_ at 37°C. The medium was refreshed every 3 days, and the cells were passaged after achieving 80% confluency. To establish a high‐dose MP environment, BMSCs were treated with 100 μM MP for different time periods according to the clinical therapeutic dose and previous studies.^7^, ^26^, ^27^, ^28^ In specific experiments, the BMSCs were pretreated with the lentiviral vector (short hairpin MAGL [shMAGL]), siRNA (siNrf2), plasmids (OV‐MAGL and OV‐Nrf2), NOX inhibitor (diphenyleneiodonium chloride [DPI], 10 μM and VAS2870, 10 μM), Nrf2 agonist (curcumin, 20 μM), Nrf2 inhibitor (ML385, 20 μM), MAGL inhibitor (MJN110, 1 μM), or 2‐arachidonoylglycerol (2AG, 20 μM) before MP administration.

### 2.7 Cell proliferation and toxicity assay

Water‐soluble tetrazolium salt‐8 was used to measure cell proliferation and toxicity. BMSCs (1 × 10^3^ cells/well in 96‐well plates) exposed to various concentrations of MP. At various time points, 100 μL of fresh medium containing 10 μL CCK8 stock solution (Beyotime Biotech, Shanghai, China) was added to each well. After 2 h, the OD value was recorded by measuring the absorbance at 450 nm.

### 2.8 Western blotting

In vitro‐cultured BMSCs were harvested and lysed in radioimmunoprecipitation assay (RIPA) buffer (NCM Biotech, Soochow, China). For the extraction of proteins from the bone, the marrow cavity was flushed with chilled phosphate‐buffered saline (PBS), and the bone tissues were minced in liquid nitrogen before the addition of RIPA buffer. Equal quantities of cell lysates were electrophoresed and transferred onto a nitrocellulose membrane. After 1 h of blocking in QuickBlock Blocking Buffer (Beyotime Biotech), the membranes were treated with primary antibodies and the corresponding secondary antibodies (1:5000, Abcam). The separated bands were visualized using chemiluminescence (Pierce ECL). The obtained autoradiograph was analyzed through optical density analysis. The relative gray values were measured using the Image Lab 3.0 software.

### 2.9 RT‐PCR

RNA was isolated from BMSCs through standard protocols. RNA concentration was determined using a NanoDrop 2000 spectrophotometer (Thermo Fisher Scientific, Waltham, MA, USA). Next, cDNA was synthesized using RNA/reverse transcriptase mixtures. PCR amplification was performed using qPCR MasterMix (Biotium) and forward/reverse primers. mRNA expression data were analyzed using the 2^−ΔΔCq^ method. Sangon Biotech (Shanghai, China) provided all primers.

### 2.10 Lentivirus transfection

A lentiviral vector [LV3 (H1/GFP&Puro)] encoding shMAGL was obtained from GenePharma (Shanghai, China). The lentiviral transfection protocol was performed as per the manufacturer's instructions. Briefly, lentiviruses with the multiplicity of infection of 100 and polybrene (5 μg/mL) were added to a 6‐well plate when BMSCs were 40%–60% confluent. MP was used to treat the cells after 72 h. The relative gene and protein expression levels of MAGL were used to evaluate the transfection efficacy.

### 2.11 RNA interference and plasmid transfection

siRNA (RNA oligo) and overexpression plasmids (pcDNA3.1) were provided by GenePharma. RNA interference and plasmid transfection protocols were performed following the manufacturer's instructions. Briefly, diluted siRNA/plasmid and GP‐transfect‐Mate were added to a 6‐well plate when BMSCs were 70% confluent. The cells were then incubated for 24 h. The relative gene and protein expression levels of MAGL were used to evaluate the transfection efficacy.

### 2.12 TUNEL assay

The TUNEL Assay Kit was purchased from Beyotime Biotech and was used to examine cell and tissue apoptosis. BMSCs cultured on glass coverslips were immersed in 4% paraformaldehyde for 30 min and then incubated in 0.3% Triton X‐100 at 25°C for 5 min, whereas for paraffin sections, the cells were incubated in proteinase K for 20 min after routine dewaxing and rehydration. Next, the samples were incubated in the TUNEL detection solution (terminal deoxynucleotidyl transferase enzyme: fluorescent marker, 1:9) for 60 min. Anti‐fluorescence quenching and sealing solution containing DAPI was used to seal the coverslips, and TUNEL‐positive BMSCs were observed under a fluorescence microscope.

### 2.13 ROS assay

ROS levels in BMSCs were evaluated using an ROS assay kit (Beyotime Biotech). After the medication interventions, BMSCs were incubated in a serum‐free medium containing 2ʹ,7ʹ‐dichlorofluorescein diacetate (1:1000) for 20 min at 37°C. The cells were then rinsed with the serum‐free medium three times. Finally, ROS‐positive BMSCs were observed under an inverted fluorescence microscope.

### 2.14 Immunofluorescence assay

First, BMSCs were washed twice with PBS. After 15 min incubation in cold paraformaldehyde, BMSCs were blocked with QuickBlockTM IF blocking solution (Beyotime Biotech) and incubated for 1 h. Next, the cells were incubated in primary antibodies (anti‐MAGL and anti‐Nrf2) overnight at 4°C. After washing twice with PBS, phalloidin and the corresponding fluorescein secondary antibodies were incubated at ambient temperature for 2 h under dark conditions. Finally, the BMSCs were stained with DAPI and intracellular protein expression was evaluated using fluorescence microscopy.

### 2.15 Statistical analysis

All in vivo and in vitro experiments were repeated at least three times. Results were assessed via analysis of variance using SPSS Version 20, and the values are presented as mean ± standard deviation. All post hoc analyses were conducted using Tukey's test. Differences were considered statistically significant at *p* < 0.05.

## 3 RESULTS

### 3.1 GCs promote apoptosis by inducing oxidative stress and upregulate MAGL expression in BMSCs

**FIGURE 1:**
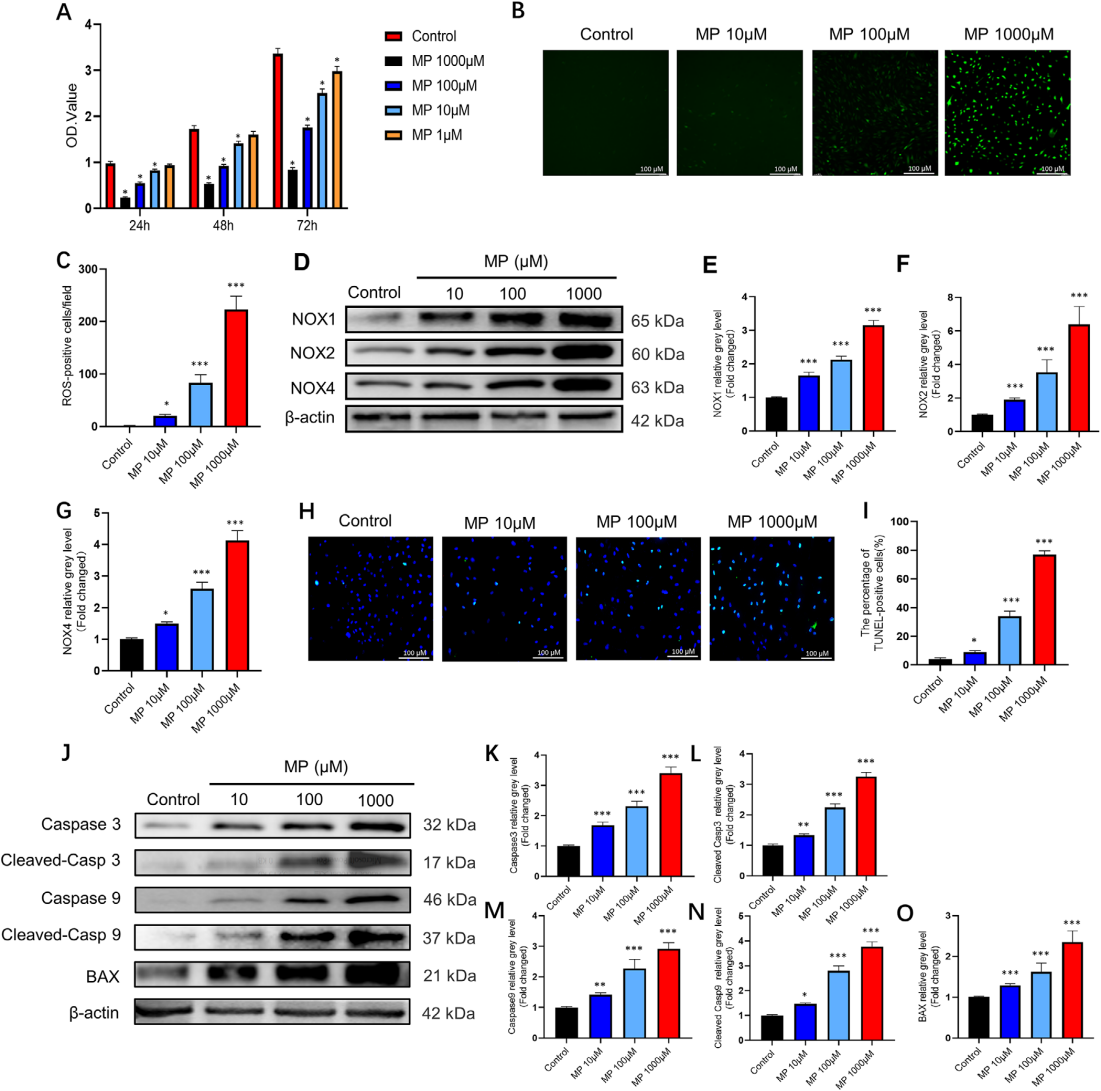
Glucocorticoids promote bone marrow mesenchymal stem cell (BMSC) apoptosis by inducing oxidative stress. (A) Cytotoxicity of methylprednisolone (MP) was assessed on BMSCs using CCK‐8 assay. (*n* = 5, mean ± SD, *p < 0.05 versus control group). (B) Reactive oxygen species (ROS) staining was performed to test the correlation between different concentrations of MP and the level of oxidative stress. (C) Average number of ROS‐positive cells per field in each group. (D–G) BMSCs were stimulated with various concentrations of MP for 24 h, the expressions of NOX1, NOX2, and NOX4 were analyzed by western blot. (H) TUNEL staining was performed to test the correlation between different concentrations of MP. (I) Quantitative analysis of the positively TUNEL‐stained BMSCs ratio in (H). (J–O) BMSCs were stimulated with various concentrations of MP for 48 h, the expression levels of the apoptosis‐related proteins were shown (*n* = 3, mean ± SD; *p < 0.05; **p < 0.01; ***p < 0.005 versus control group). These studies were performed at least three biological replicates

First, BMSCs were incubated in α‐MEM containing various concentrations of MPSS to confirm whether MP could inhibit BMSCs viability. A high dose of MP (≥1 μM) was found to be toxic to BMSCs (Figure 1A). We then tested whether MP‐induced toxicity correlated with oxidative stress. Accordingly, ROS levels were found to have a significant positive correlation with MP concentration (Figure 1B and C). NOX is an important source of ROS in cells. Western blotting results showed that the overexpression of NOX1, NOX2, and NOX4 was accompanied by an increase in MP concentration (Figure 1D–G). In addition, at high concentrations, MP induced apoptosis and a dose‐dependent increase in the expression levels of apoptosis‐related proteins (Figure 1H–O). We examined oxidative stress levels and apoptosis in BMSCs treated with an MP concentration of 100 μM at different time points. Based on the results, GC‐induced oxidative stress levels and cell apoptosis increased over time (Figure S1A–L). These results demonstrate that GCs can induce oxidative damage and cell death.

**FIGURE 2:**
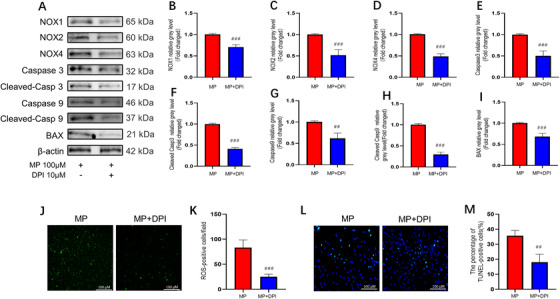
Suppression of oxidative stress alleviated bone marrow mesenchymal stem cell (BMSC) apoptosis. (A–I) The relative expressions of NADPH oxidative isozymes and apoptosis‐related proteins. In MP+MJN110 group, BMSCs were pretreated with NOX inhibitor diphenyleneiodonium chloride (DPI) (10 μΜ) for 24 h; MP (100 μΜ) was then added for 24 or 48 h. (J) Reactive oxygen species (ROS) staining of BMSCs (methylprednisolone [MP] group versus MP + DPI group). The chronology of drug intervention is the same as that in (A). (K) Average number of ROS‐positive cells per field in both groups. (L) TUNEL staining was performed to test apoptotic rate in MP and MP + DPI group. The chronology of drug intervention is the same as that in (A). (M) Quantitative analysis of the positively TUNEL‐stained BMSCs ratio in (L) (*n* = 3, mean ± SD; #*p* < 0.05; ##*p* < 0.01; ###*p* < 0.005 versus MP group). These studies were performed at least three biological replicates

**FIGURE 3:**
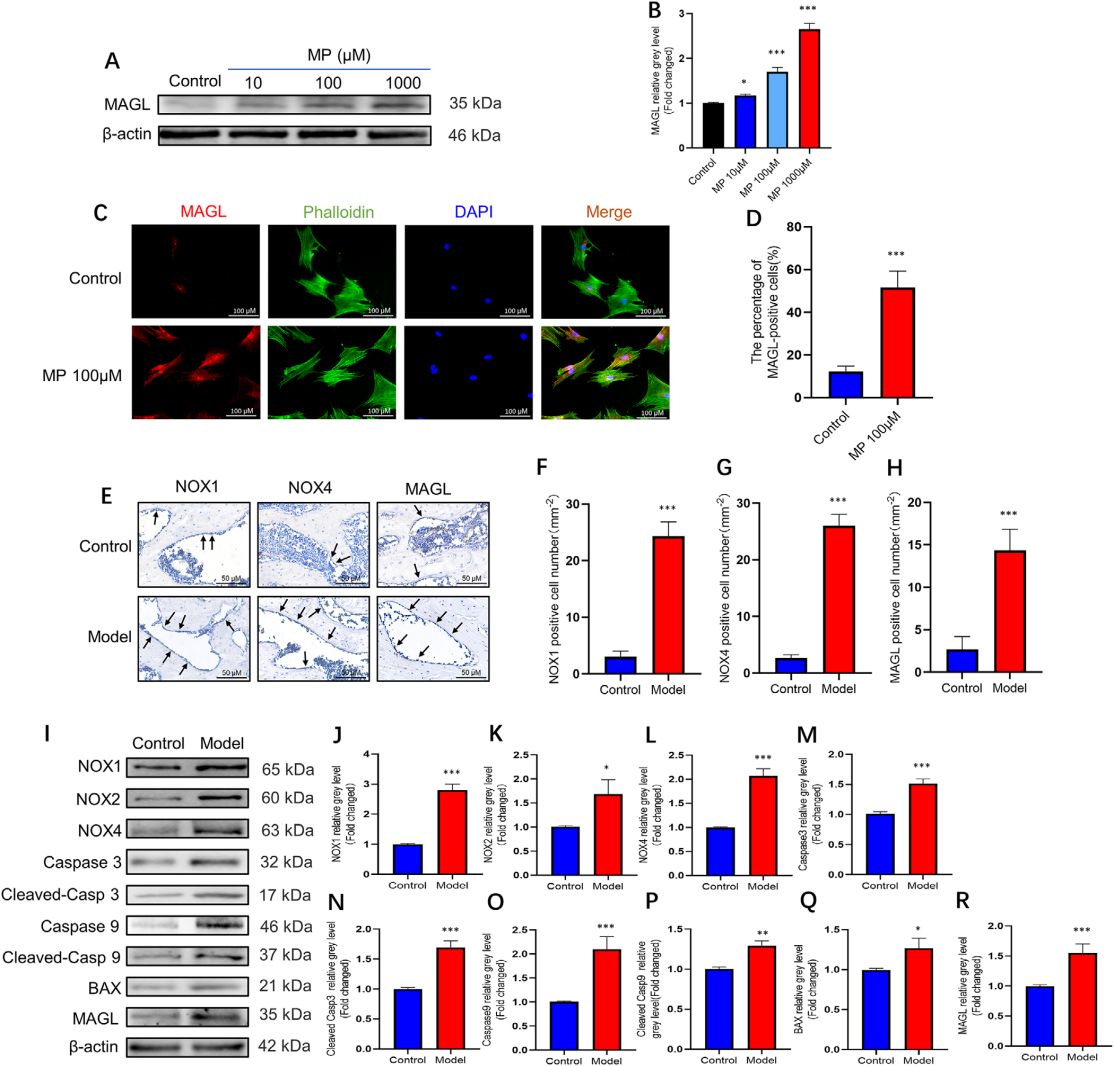
Monoacylglycerol lipase (MAGL) overexpression in bone marrow mesenchymal stem cells (BMSCs) strongly associated with glucocorticoids. (A and B) Western blot was performed to test the correlation between different concentrations of methylprednisolone (MP) and the MAGL expression level. (C) Images of immunofluorescence staining of MAGL in BMSCs after treatment with MP (100 μΜ) for 72 h. (D) Quantification of the percentage of MAGL‐positive cells (*n* = 3; mean ± SD; *p < 0.05; **p < 0.01; ***p < 0.005 versus control group). (E) IHC staining of NOX1, NOX4, and MAGL, the IHC‐positive cells were marked with black arrows. (F–H) Average number of IHC‐positive cells per field. (I–R) The protein expression levels of NADPH oxidative isozymes, apoptosis‐related proteins, and MAGL in bone tissues (control group versus MP group) (*n* = 5; mean ± SD; *p < 0.05; **p < 0.01; ***p < 0.005 versus control group). All these studies were performed at least three biological replicates

We hypothesized that oxidative stress is a major factor in GC‐induced BMSC apoptosis. Our results indicated that DPI, as an inhibitor of NOX, significantly lowered the expression levels of NOX family proteins. Next, we assessed the effect of DPI on GC‐induced apoptosis. Western blotting results showed that MP‐induced overexpression of caspase 3, cleaved caspase 3, caspase 9, cleaved caspase 9, and BAX was notably attenuated by DPI treatment (Figure 2A–I). Additionally, ROS assay and TUNEL staining showed that oxidative stress levels and cell apoptosis were significantly decreased in the DPI‐treated group compared with those in the MP‐treated group (Figure 2J–M). To confirm the reliability of these results, we repeated these experiments with another pan‐NOX inhibitor (VAS2870) and obtained similar results (Figure S2A–L). Altogether, our results indicate that oxidative stress is an important contributor to GC‐induced BMSC apoptosis. To investigate whether associations existed between the MAGL expression and MP‐induced ONFH model, we quantified MAGL expression in BMSCs via western blot analysis (Figure 3A and B). Upon MP treatment, the expression level of MAGL increased with MP concentration. Immunofluorescence results further confirmed that MAGL expression was induced by MP (Figure 3C and D).

To verify the results of these in vitro experiments, we established a GC‐induced ONFH rat model by injecting both LPS and MP. In vivo results showed that GC‐induced ONFH was successfully achieved in 75% (6/8) of rats, whereas no ONFH occurred in rats of the control group (0/8). Compared to the control group, a significant decrease in BV, BV/TV, and Tb.Th, and a significant increase in Tb.Sp were observed in the model group at 6 weeks after MP treatment. Based on micro‐CT images and the H&E staining results, we found that in the model group, the subchondral trabecular bone disappeared completely, and the levels of fat droplets, pyknotic nuclei, and empty lacunae increased significantly in the femoral head (Figure S3A–F). The TUNEL assay results revealed the presence of more apoptotic cells in the femoral head of the model group than in the control group (Figure S3G and H). More importantly, we observed more MAGL‐, NOX1‐, and NOX4‐positive cells in the femoral head sections via immunohistochemistry (Figure 3E–H). Western blotting results further confirmed that elevated MAGL protein levels were accompanied by increased expression of the associated NOX family and apoptosis‐related proteins, such as caspase 3, cleaved caspase 3, caspase 9, cleaved caspase 9, and BAX in the bone tissues of rats from the model group (Figure 3I–R). Therefore, our results confirmed that GCs not only promoted apoptosis by inducing oxidative stress but also increased MAGL expression in BMSCs.

### 3.2 MAGL blockade suppresses GC‐induced oxidative stress and BMSC apoptosis

**FIGURE 4:**
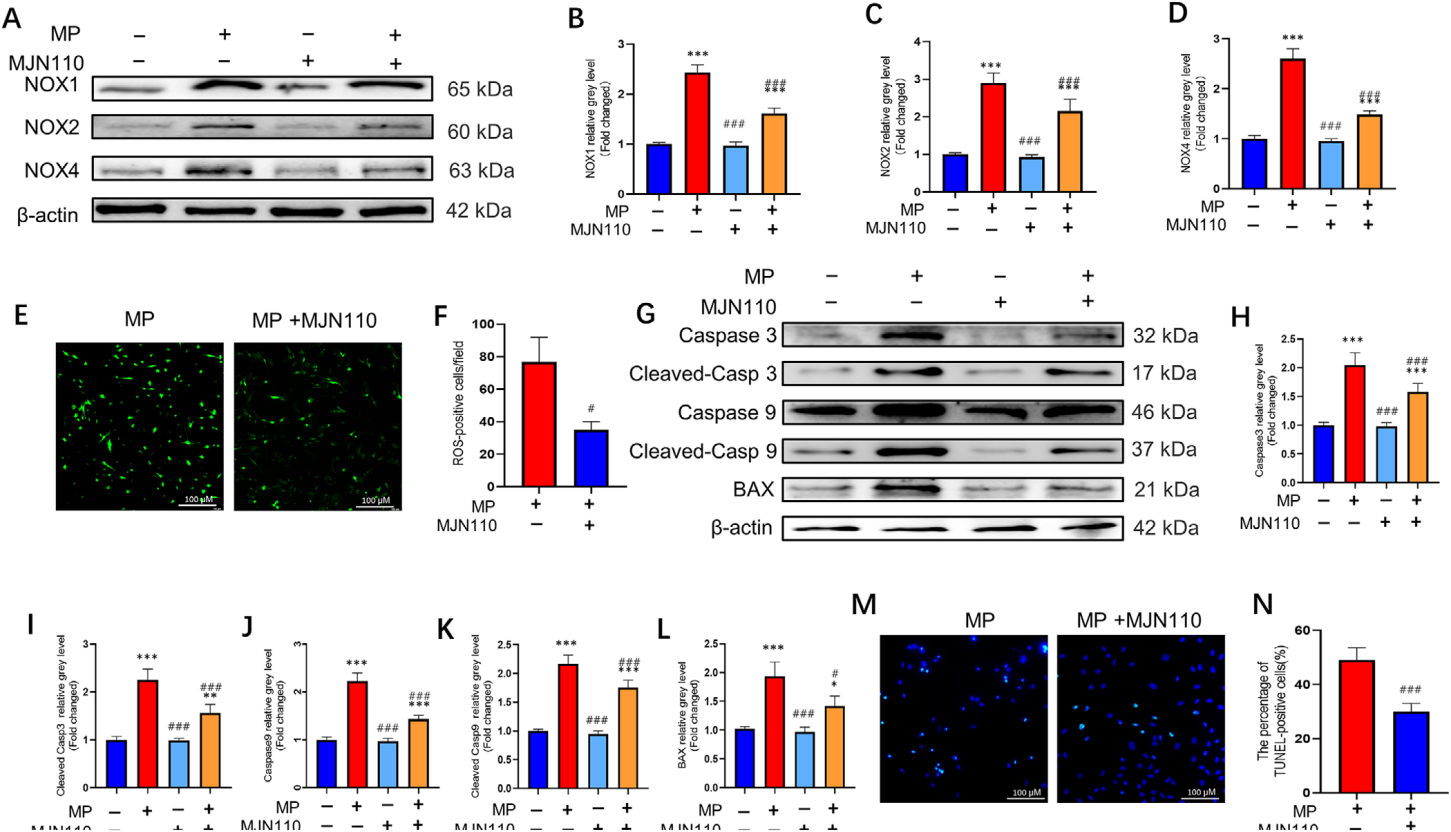
Monoacylglycerol lipase (MAGL) inhibition alleviates GC‐induced oxidative stress and apoptosis in bone marrow mesenchymal stem cells (BMSCs). (A–D) The protein expression levels of NADPH oxidative isozymes. BMSCs were pretreated with MAGL inhibitors MJN110(1 μΜ) for 24 h; Methylprednisolone (MP) (100 μΜ) was then added for 24 h. (E) Reactive oxygen species (ROS) staining of BMSCs (MP group versus MP + MJN110 group). In MP + MJN110 group, BMSCs were pretreated with MAGL inhibitors MJN110 (1 μΜ) for 24 and MP (100 μΜ) was then added for 24 h. (F) Average number of ROS‐positive cells per field in both groups. (G–L) Western blot results for the expressions of Caspase3, Cleaved Caspase3, Caspase9, Cleaved Caspase9, and BAX. BMSCs were pretreated with MAGL inhibitors MJN110 (1 μΜ) for 24 h and MP (100 μΜ) was then added for 48 h. (M) TUNEL staining was performed to test apoptotic rate in MP and MP + MJN110 group. In MP + MJN110 group, BMSCs were pretreated with MAGL inhibitors MJN110 (1 μΜ) for 24 h and MP (100 μΜ) was then added for 48 h. (N) Quantitative analysis of the positively TUNEL‐stained BMSCs ratio in (M) (*n* = 3, mean ± SD; *p < 0.05; **p < 0.01; ***p < 0.005 versus control group; #*p* < 0.05; ##*p* < 0.01; ###*p* < 0.005 versus MP group). These studies were performed at least three biological replicates

As MP upregulated MAGL expression in the GC‐induced ONFH rat model, we investigated whether MAGL inhibition could suppress MP‐induced oxidative stress and apoptosis in BMSCs. Western blotting results showed that MJN110 or shMAGL significantly inhibited MAGL expression (Figures S4A and B and S5A and B). Additionally, the elevated expression of NOX family proteins was suppressed in the MAGL‐treated group (Figure 4A–D). Intracellular ROS levels decreased after MJN110 treatment or shMAGL transfection (Figure 4E and F, Figure S5E and F). These results demonstrate that oxidative stress can be effectively suppressed by MAGL blockade. We further assessed the effect of MAGL inhibition on BMSC apoptosis. The results showed that treatment with MJN110 blocked the apoptotic pathway by inhibiting the expression of apoptosis‐related proteins in the cells (Figure 4G–L). In addition, TUNEL assay results confirmed that the number of apoptotic BMSCs decreased after MAGL blockade (Figure 4M and N, Figure S5G and H). We examined oxidative stress levels and cell apoptosis in MAGL‐overexpressing BMSCs treated with or without MP. As expected, MAGL overexpression further increased GC‐induced oxidative stress levels and apoptosis in BMSCs (Figure S5C–H). Collectively, our data demonstrate that MAGL inhibition could reverse GC‐induced oxidative stress and apoptosis in BMSCs.

### 3.3 MAGL blockade activates the Keap1/Nrf2 signaling pathway and protects BMSCs from GC‐induced oxidative stress and apoptosis

**FIGURE 5:**
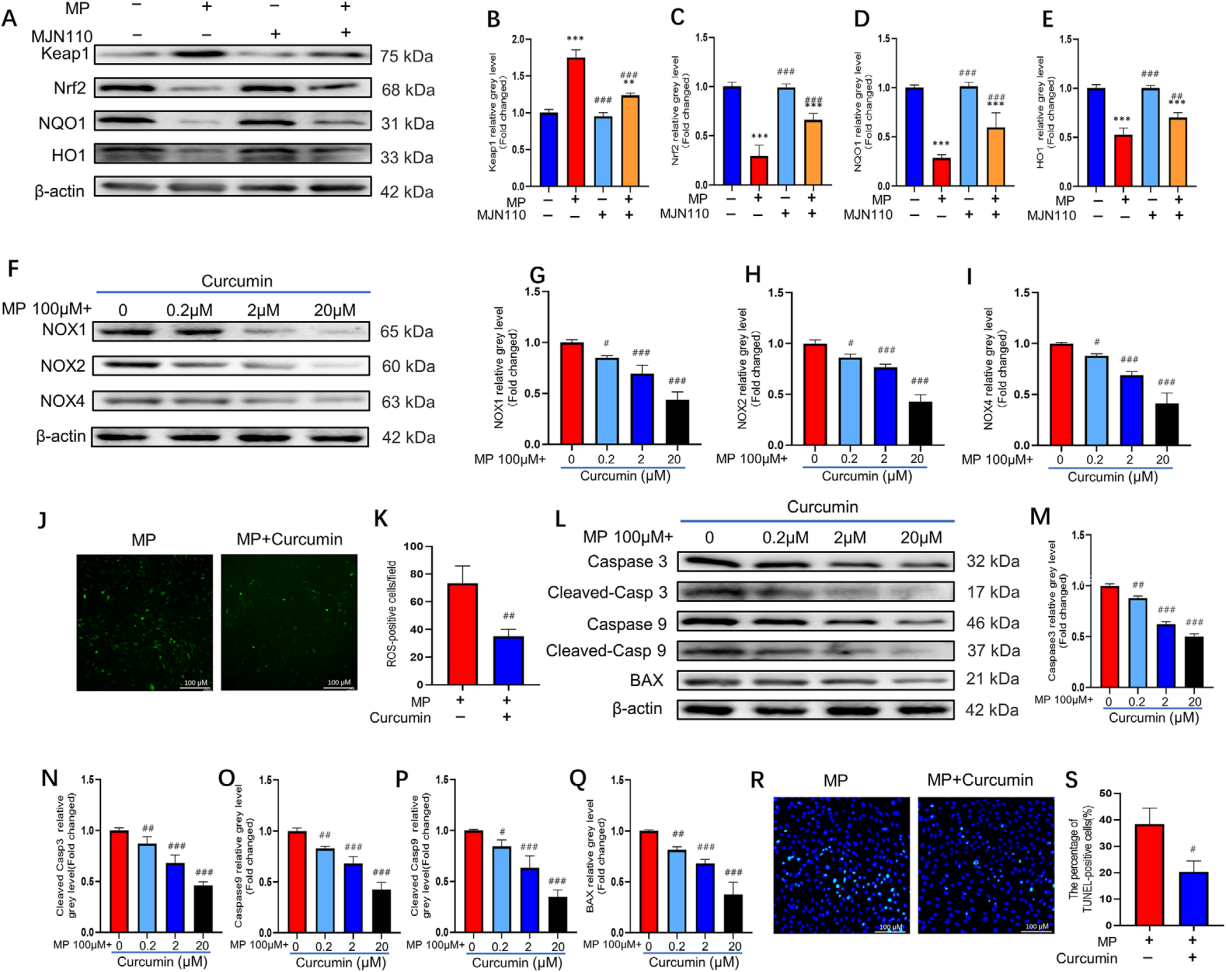
Monoacylglycerol lipase (MAGL) inhibition activates Keap1/Nrf2 signaling pathway and Nrf2 activation attenuates GC‐induced oxidative stress and apoptosis in bone marrow mesenchymal stem cells (BMSCs). (A–E) The protein expression levels of Keap1, Nrf2, NQO1, and HO1. BMSCs were pretreated with MAGL inhibitors MJN110 (1 μΜ) for 24 h; Methylprednisolone (MP; 100μΜ) was then added for 24 h. (F–I) The protein expression levels of NADPH oxidative isozymes. We preincubated BMSCs with various concentrations of curcumin for 24 h; MP (100 μΜ) was then added for 24 h. (J) ROS staining of BMSCs (MP group versus MP + curcumin group); In MP + curcumin group, we preincubated BMSCs with curcumin (20 μΜ) for 24 h, MP was then added for 24 h. (K) Average number of reactive oxygen species (ROS) positive cells per field in both groups. (L–Q) The protein expression levels of the apoptosis‐related proteins. We preincubated BMSCs with various concentrations of curcumin for 24 h, MP was then added for 48 h. (R) TUNEL staining was performed to test apoptotic rate (MP group versus MP+MJN110 group). In MP + curcumin group, we preincubated BMSCs with curcumin (20 μΜ) for 24 h, MP (100 μΜ) was then added for 24 h. (S) Quantitative analysis of the positively TUNEL‐stained BMSCs ratio in (R) (*n* = 3, mean ± SD; *p < 0.05; **p < 0.01; ***p < 0.005 versus control group; #*p* < 0.05; ##*p* < 0.01; ###*p* < 0.005 versus MP group). These studies were performed at least three biological replicates

Keap1/Nrf2 signaling is strongly correlated with oxidative stress. When activated, Nrf2 promotes the transcription of NAD(P)H dehydrogenase (quinone 1) (NQO1) and heme oxygenase 1 (HO1). Therefore, to further understand the anti‐apoptotic mechanisms of MAGL inhibition in BMSCs, we assessed whether MAGL regulates GC‐induced oxidative stress and apoptosis through the Keap1/Nrf2 pathway. First, the western blotting results revealed that Nrf2, NQO1, and HO1 expression were significantly downregulated, whereas Keap1 expression was upregulated in the MP‐treated group. Additionally, we found that treatment with MJN110 or shMAGL markedly increased Nrf2, NQO1, and HO1 expression, but inhibited Keap1 expression (Figure 5A–E, Figure S6A–E). Immunofluorescence results confirmed that MAGL blockade upregulated Nrf2 expression in MPSS‐treated BMSCs (Figure S7A and B). Next, we investigated whether elevated Nrf2 expression conferred a protective effect on MP‐treated BMSCs. The naturally occurring Nrf2 activator, curcumin, was added to the MP‐containing medium. Western blotting and immunofluorescence staining results collectively indicated that Nrf2 expression was significantly increased by curcumin treatment (Figure S8A–F). After curcumin treatment, the expression of NOX family and apoptosis‐related proteins decreased significantly (Figure 5F–I and L–Q). ROS staining and TUNEL assay results further confirmed that curcumin was effective at alleviating GC‐induced oxidative stress and apoptosis (Figure 5J, K and R, S). These experiments were repeated three times using Nrf2‐overexpressing BMSCs and similar results were observed (Figure S9A–U). These results provide evidence that the Keap1/Nrf2 pathway plays an important role in GC‐induced apoptosis.

**FIGURE 6:**
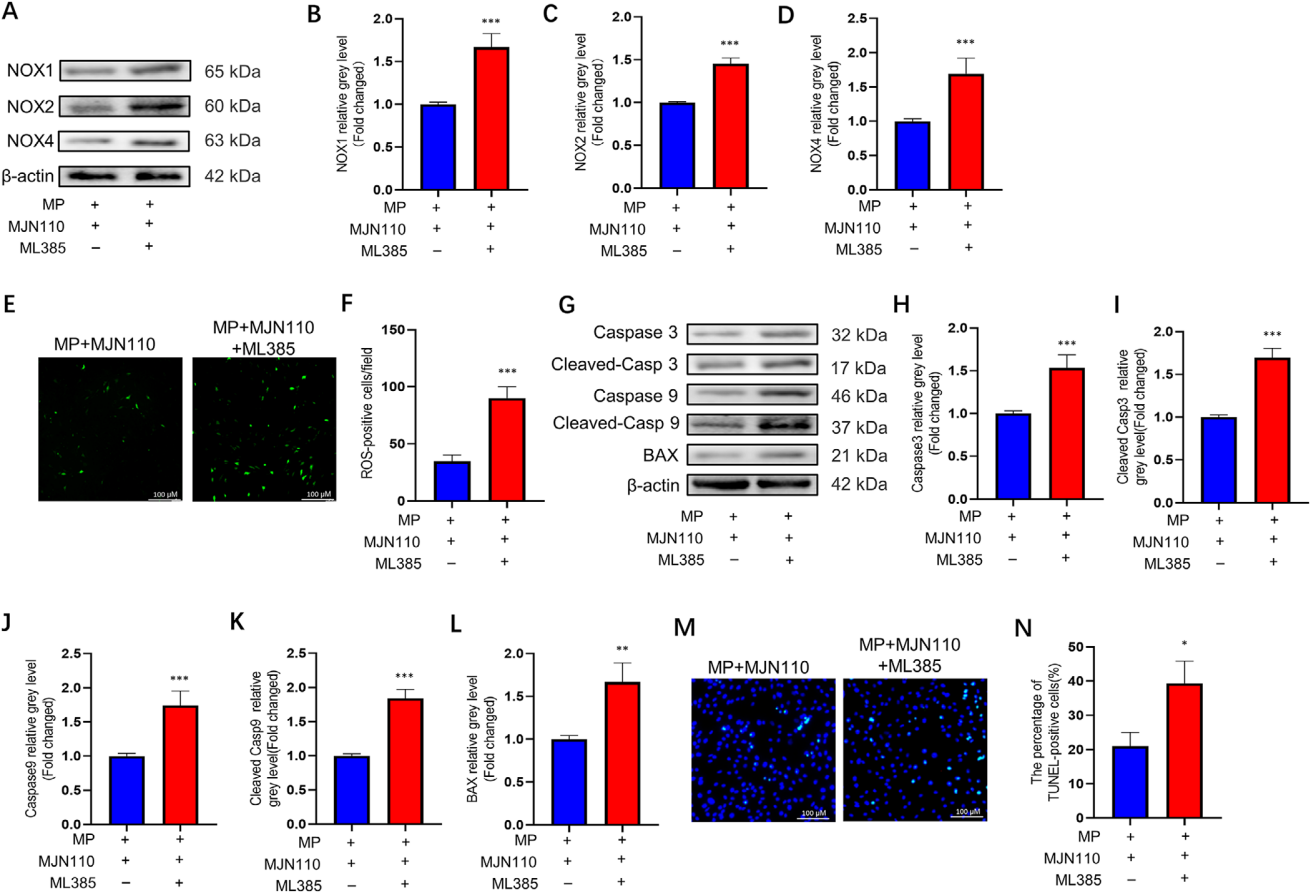
Monoacylglycerol lipase (MAGL) inhibition protects BMSCs from GC‐induced oxidative stress and apoptosis through activation of Keap1/Nrf2 cascade. (A–D) The protein expression levels of NOX1, NOX2, and NOX4. In MP + MJN110 + ML385 group, bone marrow mesenchymal stem cells (BMSCs) were pretreated with MAGL inhibitors MJN110 (1 μΜ) and ML385 (20 μΜ) for 24 h; MP (100 μΜ) was then added for 24 h. (E) ROS staining of BMSCs (MP + MJN110 group versus MP + MJN110 + ML385 group. The chronology of drug intervention is the same as that in (A). (F) Average number of reactive oxygen species (ROS) positive cells per field in both groups. (G–L) The protein expression level of Caspase3, cleaved Caspase3, Caspase9, cleaved Caspase9, and BAX. In MP + MJN110 + ML385 group, BMSCs were pretreated with MAGL inhibitors MJN110 (1 μΜ) and ML385 (20 μΜ) for 24 h; MP (100 μΜ) was then added for 48 h. (M) TUNEL staining was performed to test apoptotic rate in MP + MJN110 and MP + MJN110 + ML385 groups. The chronology of drug intervention is the same as that in (G). (N) Quantitative analysis of the positively TUNEL‐stained BMSCs ratio in (M) (*n* = 3, mean ± SD; *p < 0.05; **p < 0.01; ***p < 0.005 versus MP + MJN110 group). These studies were performed at least three biological replicates

To further verify whether the protective effect of MAGL inhibition on BMSCs was dependent on the Keap1/Nrf2 signaling pathway, we used ML385 to suppress Nrf2 expression in BMSCs. ML385 effectively downregulated Nrf2 expression (Figure S10). Western blotting and immunofluorescence staining results showed that ML385 partially restored the decreased expression of the NOX family and apoptosis‐related proteins induced by inhibiting MAGL (Figure 6A–D and G–L). Moreover, a notable increase was observed in ROS levels and cell apoptosis after ML385 treatment (Figure 6E,F and M,N). We repeated the aforementioned experiments with Nrf2‐knockdown BMSCs and obtained similar results (Figure S11A–U). These data confirm that MAGL inhibition negatively regulates GC‐induced oxidative stress and apoptosis by activating the Keap1/Nrf2 signaling pathway in BMSCs.

### 3.4 MAGL inhibition attenuates GC‐induced ONFH

**FIGURE 7:**
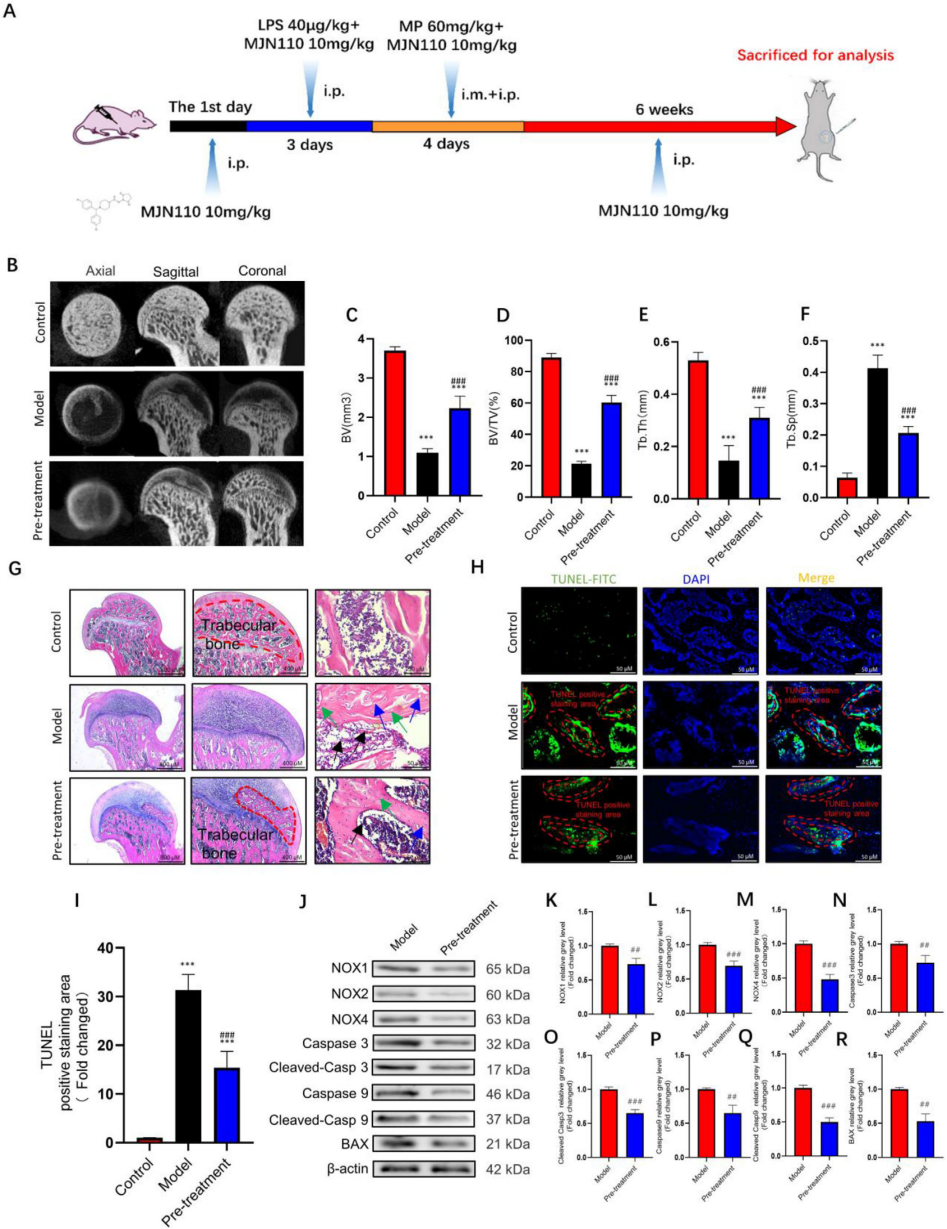
Pre‐treatment with monoacylglycerol lipase (MAGL) inhibitor alleviates GC‐induced osteonecrosis of the femoral head (ONFH). (A) The timeline of the GC‐induced ONFH model and administration of MJN110 in vivo. (B) Images of micro‐computed tomography (Micro‐CT). (C) Bone volume (BV). (D) Bone volume fraction (BV/TV). (E) Trabecular thickness (Tb.Th). (F) Trabecular spacing (Tb.Sp). (G) Hematoxylin and eosin (H&E) staining. (H) TUNEL staining of bone tissues. (I) Quantitative analysis of the area of TUNEL‐positive stain in (H). (J–R) The protein expression level of NADPH oxidative isozymes, apoptosis‐related proteins, and MAGL in bone tissues between model and pretreatment groups (*n* = 5, mean ± SD; *p < 0.05; **p < 0.01; ***p < 0.005 versus control group; #*p* < 0.05; ##*p*< 0.01; ###*p* < 0.005 versus MP group). Green arrows show nuclear pyknosis, blue arrows show empty osteocyte lacunae, and black arrows show fat droplets. All these studies were performed at least three biological replicates

Using in vivo experiments, we further investigated whether MJN110 treatment influenced the morphology of the femoral head in the early stages of ONFH. Figure 7A illustrates the process of MJN110 pre‐treatment in vivo. Micro‐CT images and H&E staining results showed that, in the pre‐treatment group, the subchondral trabecular bone was partially recovered, the trabecular bones were thicker, and their alignment was more regular. Additionally, we observed that MJN110 pretreatment significantly reduced the number of lipid droplets, pyknotic nuclei, and empty lacunae in the femoral head (Figure 7B. and G). The results of micro‐CT analysis further validated that MJN110 pretreatment not only increased the BV, BV/TV, and Tb.Th values, but also significantly decreased the Tb.Sp values in the pretreatment group at 6 weeks after MP treatment compared to values in the model group (Figure 7C–F). TUNEL assay results showed that the pre‐treatment group had fewer apoptotic cells than the model group (Figure 7H and I). According to the aforementioned histological analyses, ONFH incidence was lower in the pretreatment group than in the model group (2/8 vs. 6/8, respectively). Moreover, through western blotting and immunohistochemical staining, we further confirmed the potential of MAGL inhibition to negatively regulate oxidative stress response and cell apoptosis via the Keap1/Nrf2 pathway (Figure 7J–R, Figure S12A–G).

### 3.5 MAGL blockade improves ONFH even after the initiation of GC‐induced oxidative stress

**FIGURE 8:**
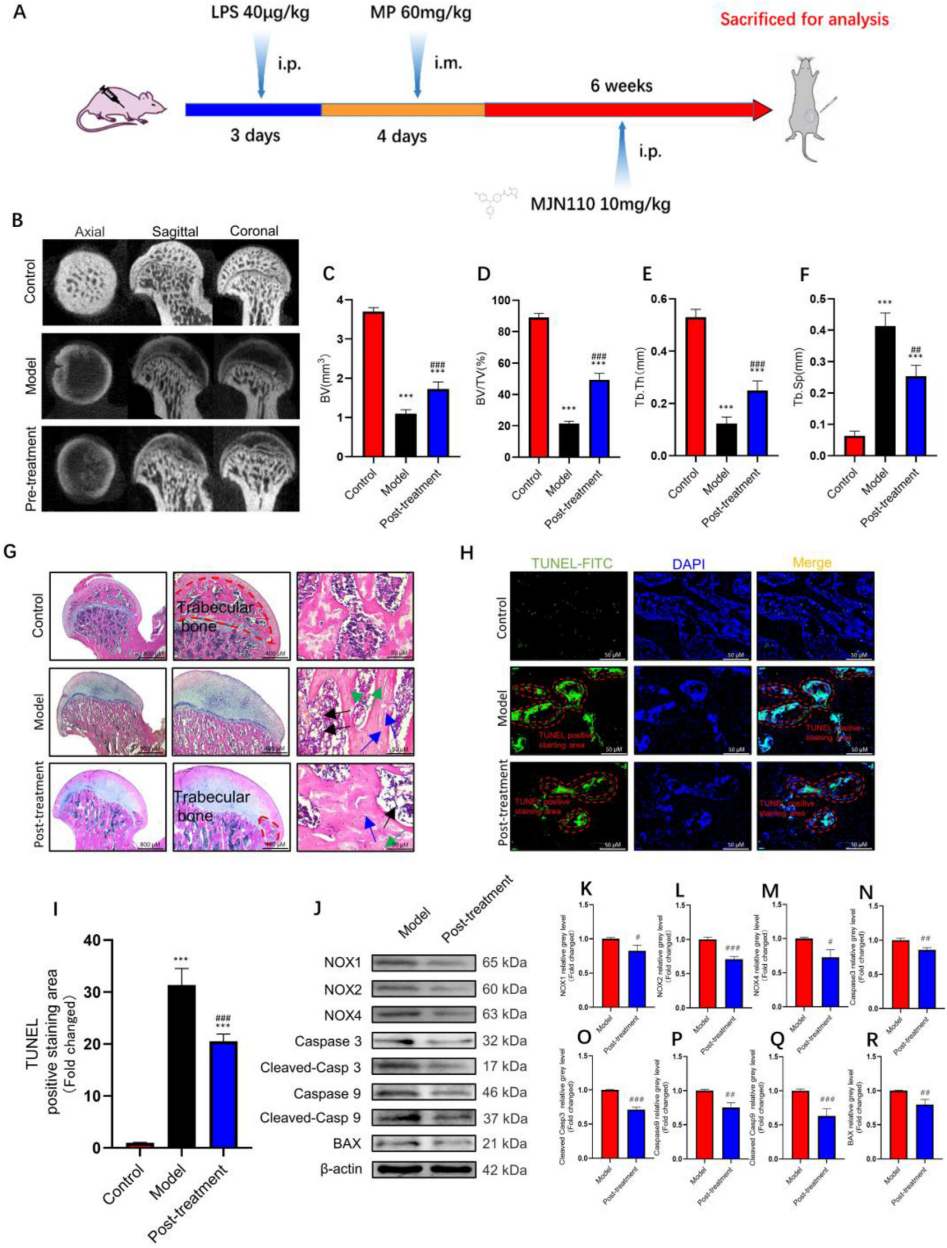
Monoacylglycerol lipase (MAGL) inhibitor can still alleviate osteonecrosis of the femoral head (ONFH) even after the initiation of GC‐induced oxidative stress. (A) The timeline of the GC‐induced ONFH model and administration of MJN110 in vivo. (B) Images of micro‐computed tomography (Micro‐CT). (C) Bone volume (BV). (D) Bone volume fraction (BV/TV). (E) Trabecular thickness (Tb.Th). (F) Trabecular spacing (Tb.Sp). (G) Hematoxylin and eosin (H&E) staining. (H) TUNEL staining of bone tissues. (I) Quantitative analysis of the area of TUNEL‐positive stains in (H). (J–R) The protein expression level of NADPH oxidative isozymes, apoptosis‐related proteins, and MAGL in bone tissues between model and posttreatment groups (*n* = 5, mean ± SD; *p < 0.05; **p < 0.01; ***p < 0.005 versus control group; #*p* < 0.05; ##*p* < 0.01; ###*p* < 0.005 versus MP group). Green arrows show nuclear pyknosis, blue arrows show empty osteocyte lacunae, and black arrows show fat droplets. All these studies were performed at least three biological replicates

**FIGURE 9:**
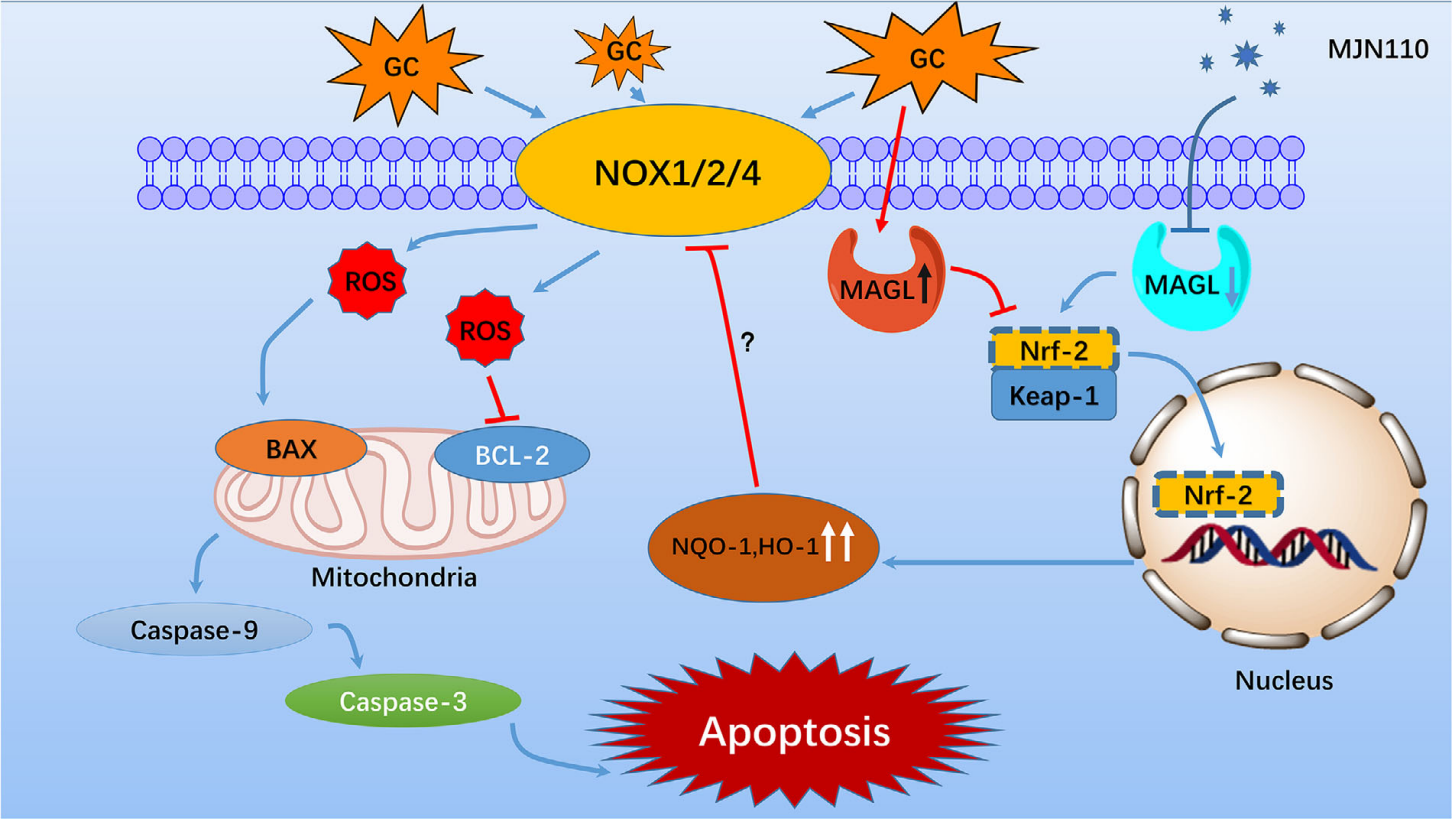
Schematic illustration of monoacylglycerol lipase (MAGL) inhibition‐mediated protection from GC‐induced oxidative stress damage. MAGL inhibition suppresses GC‐induced NADPH oxidative isozymes upregulation through activation of Keap1/Nrf2 pathway. Reduced intracellular ROS production results in a blockade of the mitochondrial apoptosis pathway

Finally, we tested whether MAGL inhibition exerted a therapeutic effect on GC‐induced ONFH. Figure 8A shows the specimen from the posttreatment group in vivo. Surprisingly, we found that although the first administration time of MJN110 was notably delayed, the subchondral trabecular bone was still partially restored (Figure 8B–G). Moreover, compared with those in the model group, there were few TUNEL‐positive BMSCs in the femoral head from the posttreatment group (Figure 8H–I). ONFH incidence in the posttreatment and model groups was estimated to be 4/8 and 6/8, respectively. Immunohistochemical staining and western blotting results further confirmed that MAGL blockade could protect BMSCs against oxidative stress and apoptosis via the Keap1/Nrf2 pathway, even after the femoral head was exposed to high doses of GC (Figures 8J–R and 9, Figure S13A–G). Overall, our results suggest that MAGL blockade not only contributes to ONFH prevention but also plays a critical role in therapy.

## 4 DISCUSSION

Increasing evidence suggests that several diseases can be effectively treated by modulating endocannabinoids.^29^, ^30^, ^31^, ^32^, ^33^ To determine the therapeutic potential of the endocannabinoid system, researchers have explored noncannabinoid receptor 1 (CB1) and non‐CB2 receptor targets, such as MAGL.^33^, ^34^, ^35^, ^36^ As a key node in the endocannabinoid system, MAGL is primarily responsible for the activation of CB2 receptor and hydrolysis of 2AG. Previous studies have shown that ischemic reperfusion injury of the liver, lungs, and kidneys is accompanied by crosstalk between MAGL and oxidants.^20^, ^37^, ^38^ Recent studies have shown that 2AG hydrolysis by MAGL controls the mutual regulation between arachidonic acid (AA) and NOX.^39^, ^40^ These findings suggest a unique interaction between MAGL and intracellular ROS accumulation.

The pathological processes underlying GC‐induced ONFH have not yet been defined. An appropriate animal model is essential for investigating the molecular and cellular mechanisms underlying GC‐induced ONFH. Rats are considered cost effective for establishing a GC‐induced ONFH animal model; however, standard induction protocols have not yet been established. Zheng et al.^41^ successfully induced ONFH in rats by pulsing injections of LPS and MP; however, animal mortality rates increased to more than 15%. Therefore, we modified the dosing regimen to reduce the mortality rate. Fortunately, none of the rats (0/8) died, and typical ONFH symptoms were observed in 75% of the rats (6/8) in the model group. These results confirmed that our GC‐induced ONFH rat model may be an ideal preclinical animal model.

Numerous studies have shown that the destructive mechanism through which GCs act on maintaining bone homeostasis is highly complex.^42^, ^43^, ^44^ GCs can not only modulate bone marrow stem cell differentiation but also increase oxidative stress levels in osteoblasts.^45^, ^46^ The NOX family plays a key role in oxidant responses. When NOX1 and NOX2 are activated, the overproduction of superoxide leads to cell apoptosis.^47^, ^48^, ^49^ NOX4 is primarily responsible for H_2_O_2_ production, and an increase in H_2_O_2_ levels induces mtDNA damage, mitochondrial protein oxidation, and mitochondrial dysfunction.^47^, ^48^, ^49^ Consistent with the results of previous reports, our results confirm that GCs activate the NOX isozyme family proteins and increase ROS levels in BMSCs. Notably, we found that NOX inhibition effectively reduced the rate of BMSC apoptosis. These results indicate that the use of antioxidants could be an effective treatment strategy for preventing GC‐induced ONFH.

As MAGL inhibition exerts antioxidative effects on multiple organs, we hypothesized that MAGL inhibition could reduce GC‐induced BMSC apoptosis by inhibiting NOX activation. As expected, both in vitro and in vivo experiments demonstrated that the functional expression of MAGL was positively correlated with MP dosage. Furthermore, MAGL blockade, using the targeted inhibitor, MJN110, or shMAGL, inhibited the expression of NOX family proteins and ROS production. Moreover, we found that MAGL blockade further reduced BAX expression and inhibited caspase 9 and caspase 3 activities, thereby alleviating apoptosis. Notably, our in vivo experiments confirmed that MAGL blockade improved the parameters of trabecular bone microarchitecture even after GC‐induced oxidative damage was initiated. These results imply that MAGL blockade may be a novel target for attenuating GC‐induced ONFH by reducing oxidative damage in BMSCs.

Nrf2, a major regulator of intracellular antioxidants, can directly reduce ROS generation by increasing the levels of ROS‐scavenging enzymes, or by indirectly inhibiting NOX activation by increasing the expression of downstream targets, such as NQO1 and HO1.^50^, ^51^, ^52^, ^53^ The NADPH/NADP ratio is downregulated by a significant upregulation of NQO1 and HO1, which then leads to a reduction in NOX activity.^54^, ^55^ In addition, products of HO1 metabolism, namely, biliverdin and CO, are potent antioxidants.^56^ GC suppresses Nrf2 transcription, whereas Nrf2 activation can significantly reduce GC‐induced oxidative stress in osteoblasts.^57^ Our results showed that MP blocked the Keap1/Nrf2 antioxidant signaling pathway, and Nrf2 activation significantly decreased ROS levels by inhibiting the expression of NOX family proteins and reducing cell apoptosis.

Western blotting results confirmed that MJN110 weakened GC‐induced Nrf2 inhibition and Keap1 activation, suggesting that Nrf2 is a target of MAGL. MAGL inhibition leads to 2AG accumulation and mimics the synaptic activation of the CB2 receptor.^58^ Interestingly, we also found that 2AG administration could rescue MP‐induced cell oxidative damage similar to what was observed for MJN110 (Figure S14A–L). Therefore, these results provide reliable evidence that MAGL blockade likely leads to Keap1/Nrf2 pathway activation via the CB2 receptor. CB2 is a core member of the endocannabinoid system, which plays an important role in a variety of ailments by affecting several signaling pathways. Several studies have confirmed that 2AG can significantly enhance extracellular signal‐regulated kinase 1/2 (ERK1/2) phosphorylation by activating the CB2 receptor.^59^, ^60^, ^61^ Under oxidative stress, ERK1/2 phosphorylation upregulates Nrf2 expression by binding NRF2 to the antioxidant responsive element.^62^, ^63^ Contrastingly, Liu et al.^64^ found that CB2 activation downregulates SP1 expression in breast cancer. SP1 can bind to the Keap1 promoter (−160/−153) and induce Keap1 transcription.^65^, ^66^, ^67^, ^68^ Moreover, AA, which is the hydrolytic product of 2AG, efficiently stimulated SP1 phosphorylation.^69^ Therefore, it is not surprising that CB2 agonists can effectively alleviate oxidative stress and inflammatory responses by activating the Nrf2 pathway.^70^, ^71^ Combining these results and previous reports, we anticipate that CB2 agonists may be beneficial for treating GC‐induced ONFH.

Furthermore, our results showed that the Nrf2 inhibitor, ML385, counteracted the protective effects of MJN110 on BMSCs. This further indicates that MAGL inactivation inhibits oxidative stress by regulating the Keap1/Nrf2 pathway and preventing GC‐induced BMSC apoptosis. However, further investigation is necessary to determine whether SP1 activation, ERK1/2 phosphorylation, and CB2 receptor play roles in MAGL blockage‐mediated regulation of Nrf2 expression. Lastly, apart from the Keap1/Nrf2 signaling pathway, other signaling pathways and abnormal lipid metabolism associated with GC‐induced ONFH cannot be ignored and should be explored in the future.

## 5 CONCLUSION

In conclusion, we confirmed that MAGL expression is positively correlated with GC‐induced ONFH. Pharmacological blockade of MAGL can effectively antagonize GC‐induced oxidative stress and apoptosis in BMSCs by activating the Keap1/Nrf2 pathway and partially alleviating ONFH. Therefore, MAGL and its inhibitors may be new generation targets for the prevention and treatment of GC‐induced ONFH. However, additional in vivo experiments and clinical data are required to validate our findings.

## CONFLICT OF INTEREST

The authors declare that they have no conflict of interest.

## Supporting information

Supporting Information

## Data Availability

The data that support the findings of this study are available from the corresponding author upon reasonable request.
