# Supplementary material for: Inhibition of MAGL activates the Keap1/Nrf2 pathway to attenuate glucocorticoid‐induced osteonecrosis of the femoral head
Source: Clin Transl Med. 2021 Jun 1;11(6):e447. doi: 10.1002/ctm2.447 (PMC8167863; doi:10.1002/ctm2.447)
Supplement: Supplementary file 1 — Supporting Information [file CTM2-11-e447-s001.pdf]

Supplementary Materials

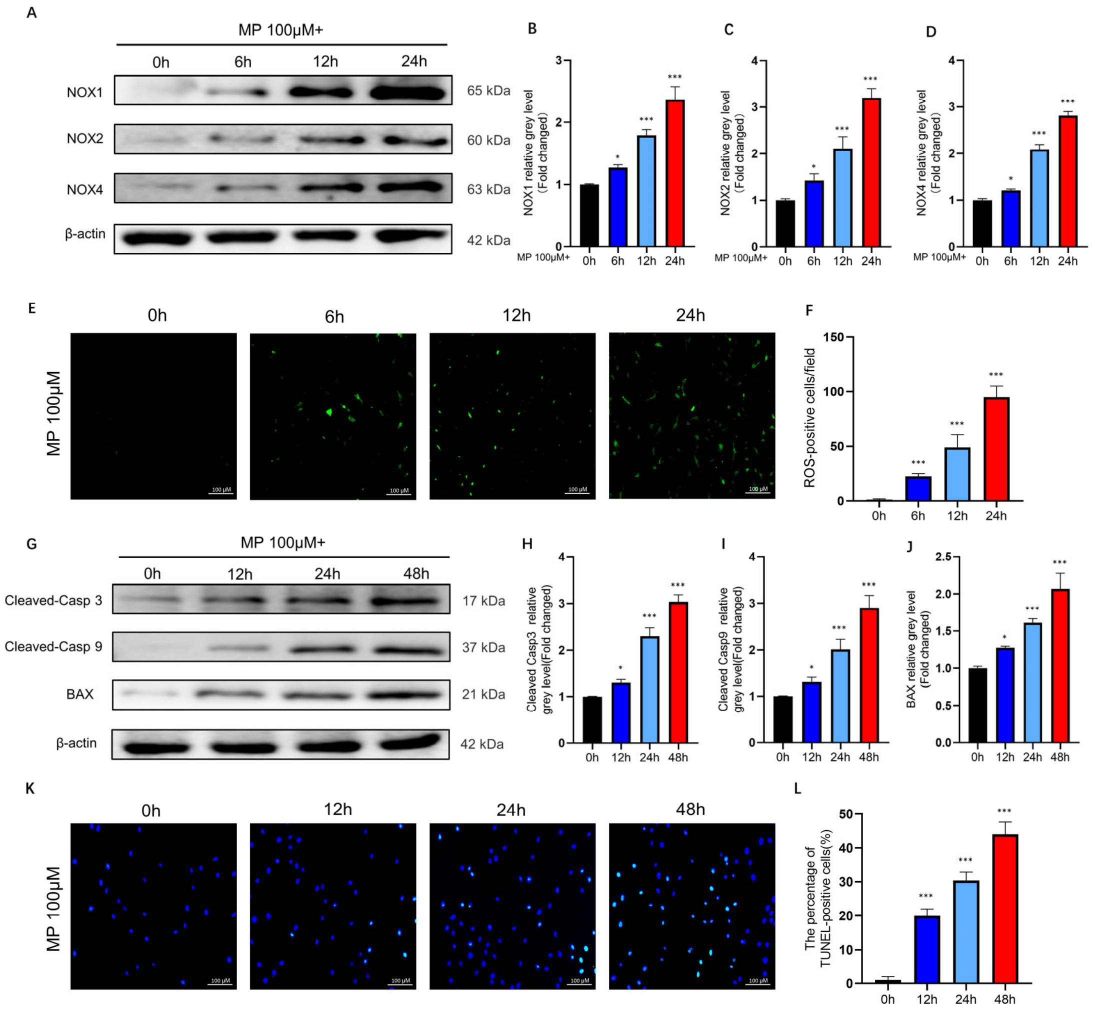

Figure S1: Glucocorticoid-induced oxidative stress and apoptosis increased over time in BMSCs. (A-D) BMSCs were stimulated with MP (100μM) for 0h, 6h, 12h or 24h, the expressions of NOX1, NOX2 and NOX4 were analyzed by western blot. (E) ROS staining was performed to test the correlation between different concentrations of MP and the level of oxidative stress. (F) Average number of ROS-positive cells per field in each group. (G-J) BMSCs were stimulated with MP (100μM) for 0h, 12h, 24h or 48h, the expressions of the apoptosis-related proteins were analyzed by western blot. (K) TUNEL staining was performed to test the correlation between different concentrations of MP. (L) Quantitative analysis of the positively TUNEL-stained BMSCs ratio in (H). (n=3, mean±S.D; *, $P<0.05$; **, $P<0.01$; ***, $P<0.005$ versus 0h group). These studies were performed at least 3 biological replicates.

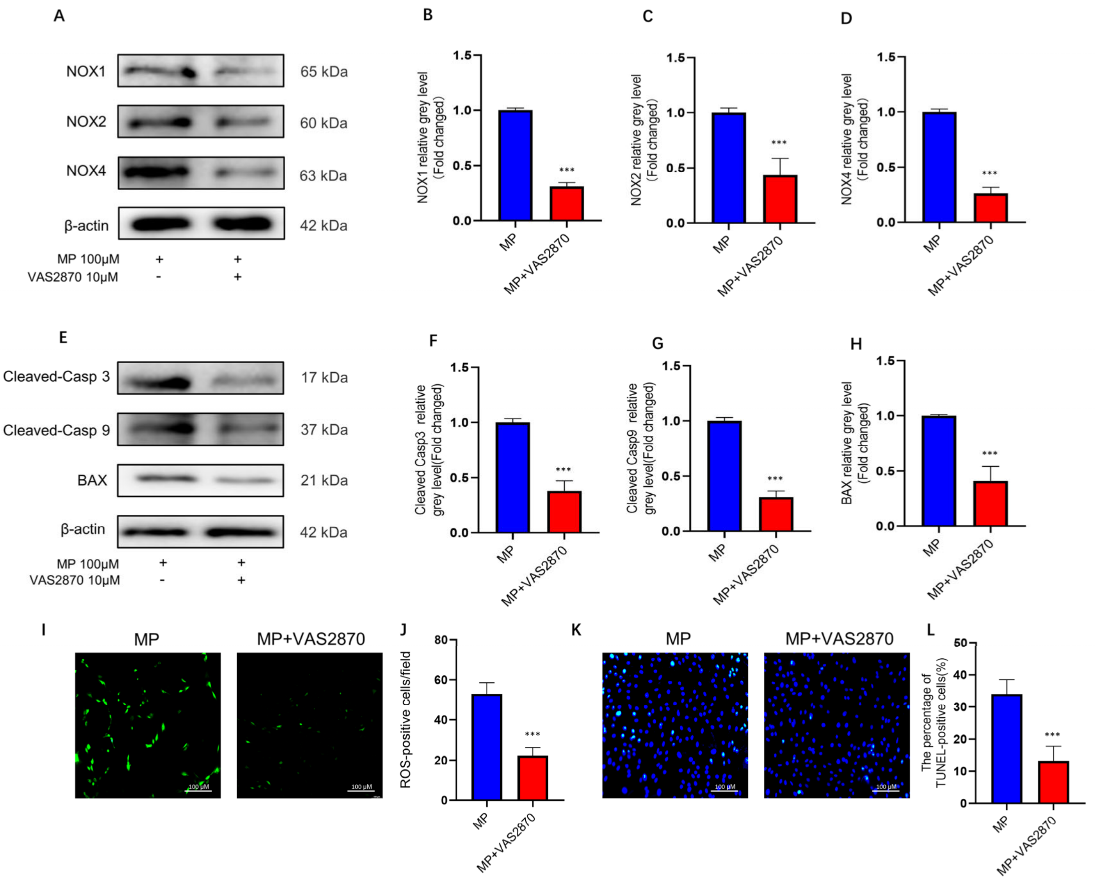

Figure S2: VAS2870 alleviated BMSC apoptosis by inducing oxidative stress. (A-H) Western blot results for the expressions of NADPH oxidative isozymes, apoptosis-related proteins. In MP+VAS2870 group, BMSCs were pretreated with NOX inhibitor VAS2870 (10μM) for 24 h; MP (100μM) was then added for 24 h or 48 h. (I) ROS staining of BMSCs (MP group versus MP +VAS2870 group). Chronology of drug intervention are the same as that in (A). (J) Average number of ROS positive cells per field in both groups. (K) TUNEL staining was performed to test apoptotic rate in MP group and MP+VAS2870 group. Chronology of drug intervention are the same as that in (A). (L) Quantitative analysis of the positively TUNEL-stained BMSCs ratio in (K). (n=3, mean ± S.D; **P*<0.05; ***P*<0.01, ****P*<0.005 versus MP group). These studies were performed at least 3 biological replicates.

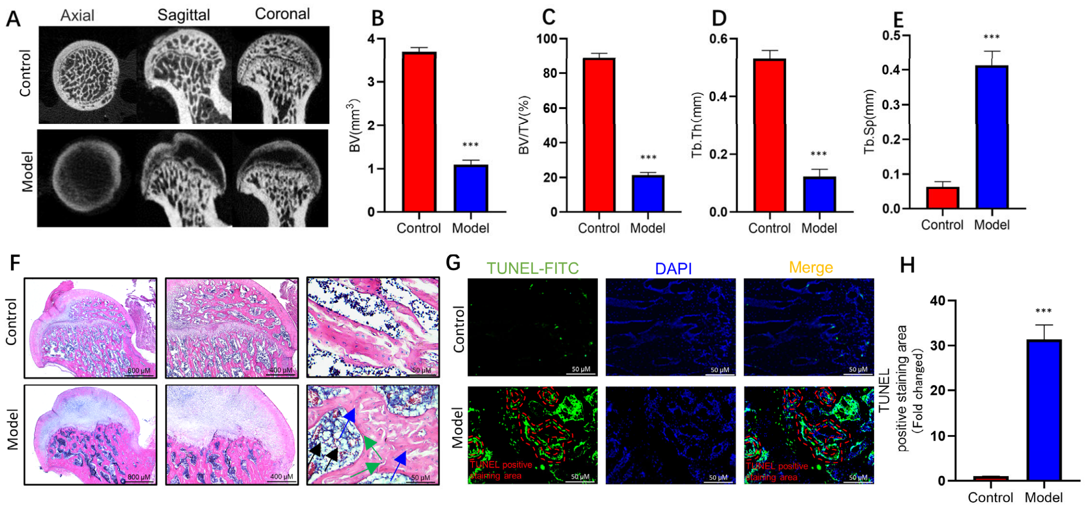

Figure S3: Characterization of GC-induced ONFH rat model. (A) Images of Micro-CT. (B) BV. (C) BV/TV. (D) Tb.Th. (E) Tb.Sp. (F) H&E staining. (G) TUNEL staining of bone tissues. (H) Quantitative analysis of the area of TUNEL-positive stains. (n=5, mean ± S.D; *, P<0.05; **, P<0.01; ***, P<0.005 versus control group). Green arrows show nuclear pyknosis, blue arrows show empty osteocyte lacunae, black arrows show fat droplets. These studies were performed at least 3 biological replicates.

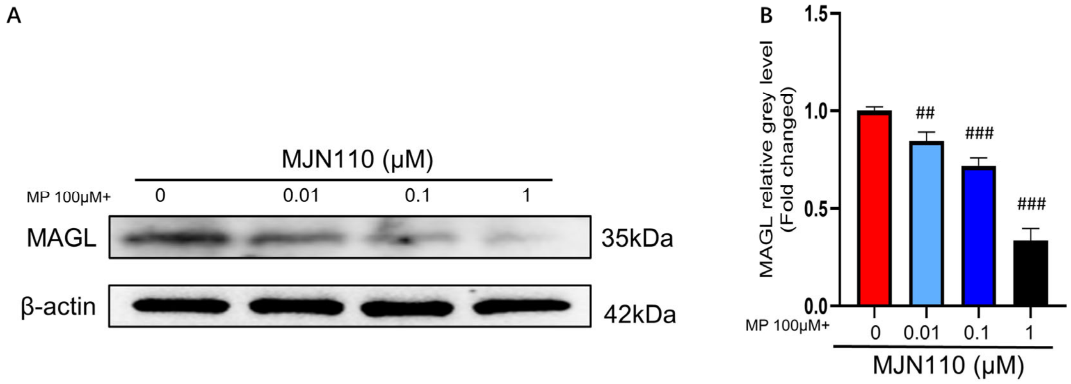

Figure S4: The effect of MJN110 on the protein expressions of MAGL. (A-B) Western blot results for the expressions of MAGL. BMSCs were first incubated for 24h with MJN110 at various concentrations; MP (100μM) was then added for 24 h. (n=3, mean ± S.D; #, P<0.05; ##, P<0.01; ###, P<0.005 versus MP group). This study was performed at least 3 biological replicates.

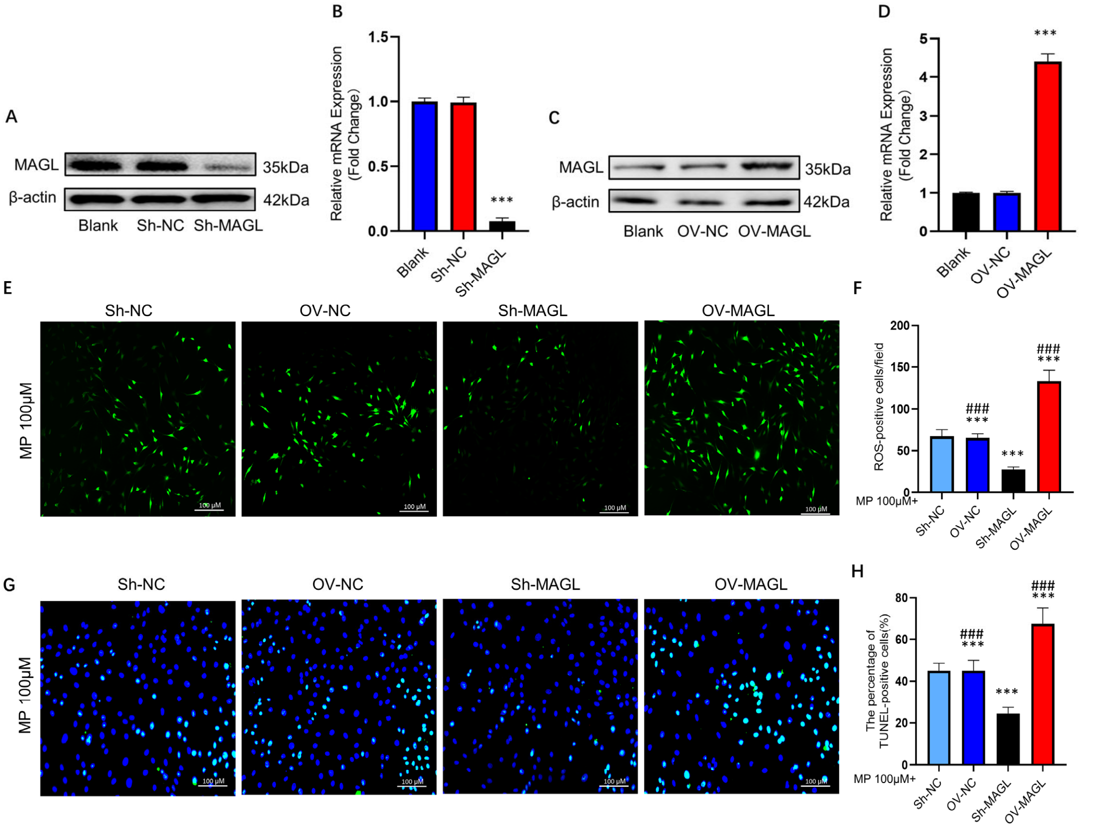

Figure S5: The effect of MAGL overexpression or knockdown on GC-induced oxidative stress and apoptosis in BMSCs. (A-B) The expression of MAGL was knocked down by lentiviral shRNA (Sh-MAGL group) compared with blank group and negative control (Sh-NC) group. (C-D) The expression of MAGL increased in MAGL plasmids group (OV-MAGL group) compared with blank group and negative control (OV-NC) group. (n=3, mean ± S.D; *P<0.05; **P<0.01; ***P<0.005 versus blank group). (E) ROS staining of BMSCs in each group. (F) Average number of ROS positive cells per field in each group. (G) TUNEL staining was performed to test the apoptotic rate in every group. (H) Quantitative analysis of the positively TUNEL-stained BMSCs ratio. (n=3, mean ± S.D; *P<0.05; **P<0.01; ***P<0.005 versus Sh-NC group. #, P<0.05; ##, P<0.01; ###, P<0.005 versus Sh-MAGL group). These studies were performed at least 3 biological replicates.

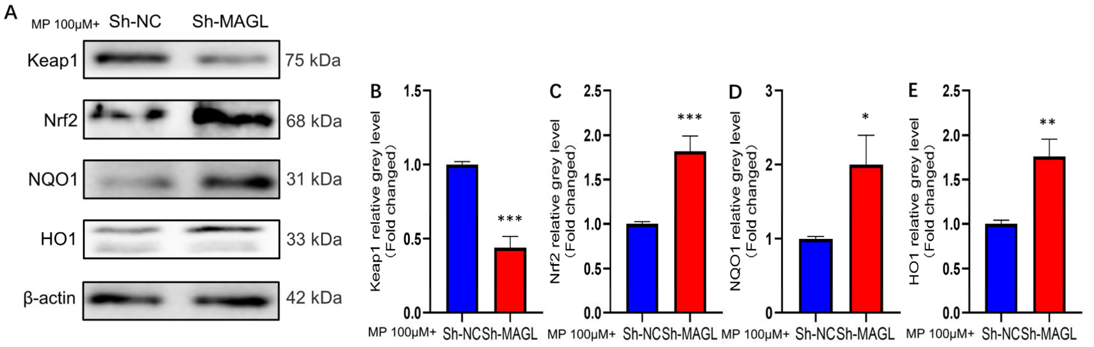

Figure S6: MAGL knockdown activates Keap1/Nrf2 signaling pathway against GC in BMSCs. (A-E) Western blot results for the expressions of Keap1, Nrf2, NQO1 and between Sh-NC group and Sh-MAGL group in BMSCs. (n=3, mean ± S.D; *P<0.05; **P<0.01; ***P<0.005 versus Sh-NC group). This study was performed 3 biological replicates.

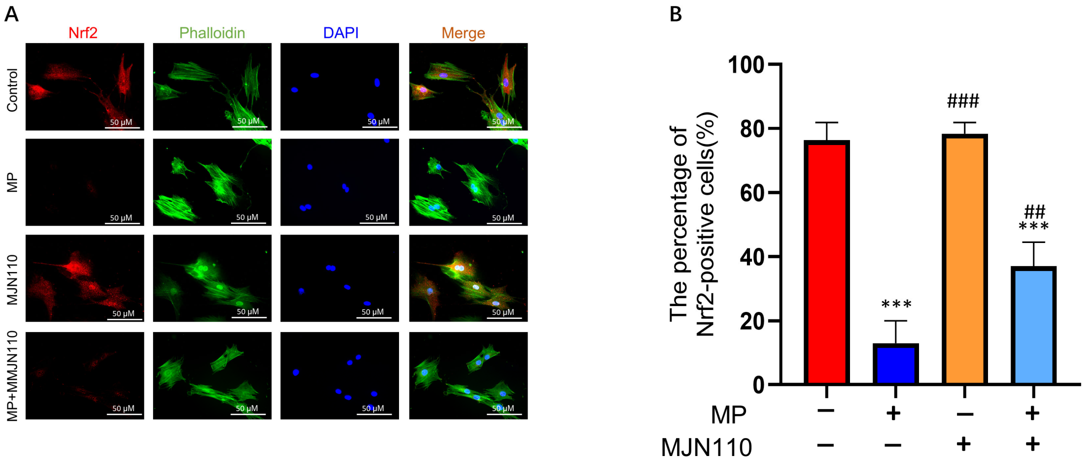

Figure S7: The effect of MJN110 on the expressions of Nrf2 in BMSCs. (A) Images of immunofluorescence staining of Nrf2 in MP (100μM) group and MP (100μM) + MJN110 (1μM) group. BMSCs were first incubated for 24h with MJN110; MP (100μM) was then added for 24 h. (B) Quantification of the percentage of Nrf2-positive cells. (n=3, mean ± S.D; **, P<0.01; ***, P<0.005 versus control group; ##, P<0.01; ###, P<0.005 versus MP group). This study was performed at least 3 biological replicates.

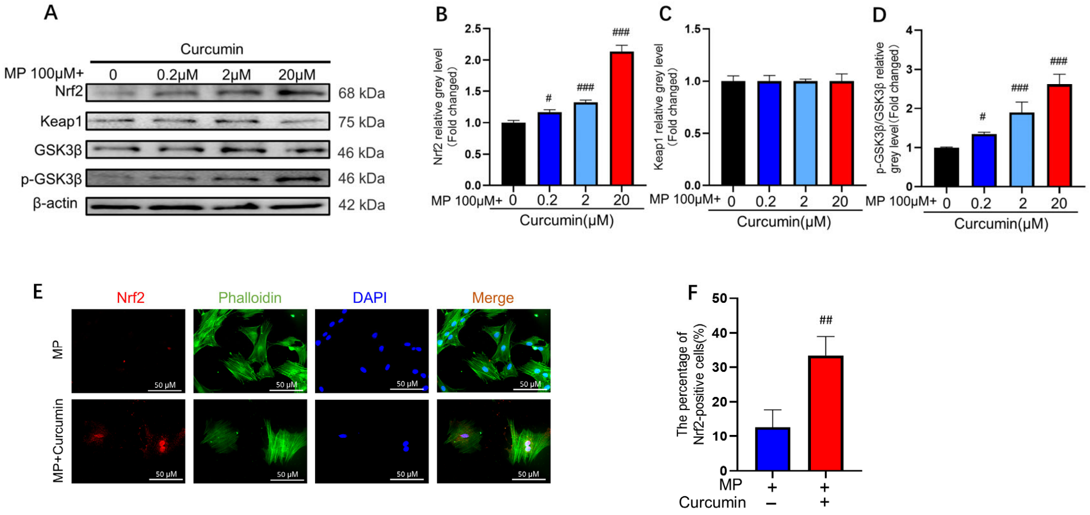

Figure S8: Curcumin promoted Nrf2 expression in BMSCs. (A-D) Western blot results for the expressions of Nrf2, Keap1, Gsk3β and p-Gsk3β. We preincubated BMSCs with various concentrations of Curcumin for 24h, MP (100μM) was then added for 24h. (E) Images of immunofluorescence staining of Nrf2 in MP group and MP+Curcumin group. In MP+Curcumin group, we preincubated BMSCs with Curcumin (20μM) for 24h, MP (100μM) was then added for 24h. (F) Quantification of the percentage of Nrf2-positive cells. (n=3, mean ± S.D; #, P<0.05; ##, P<0.01; ###, P<0.005 versus MP group). This study was performed at least 3 biological replicates.

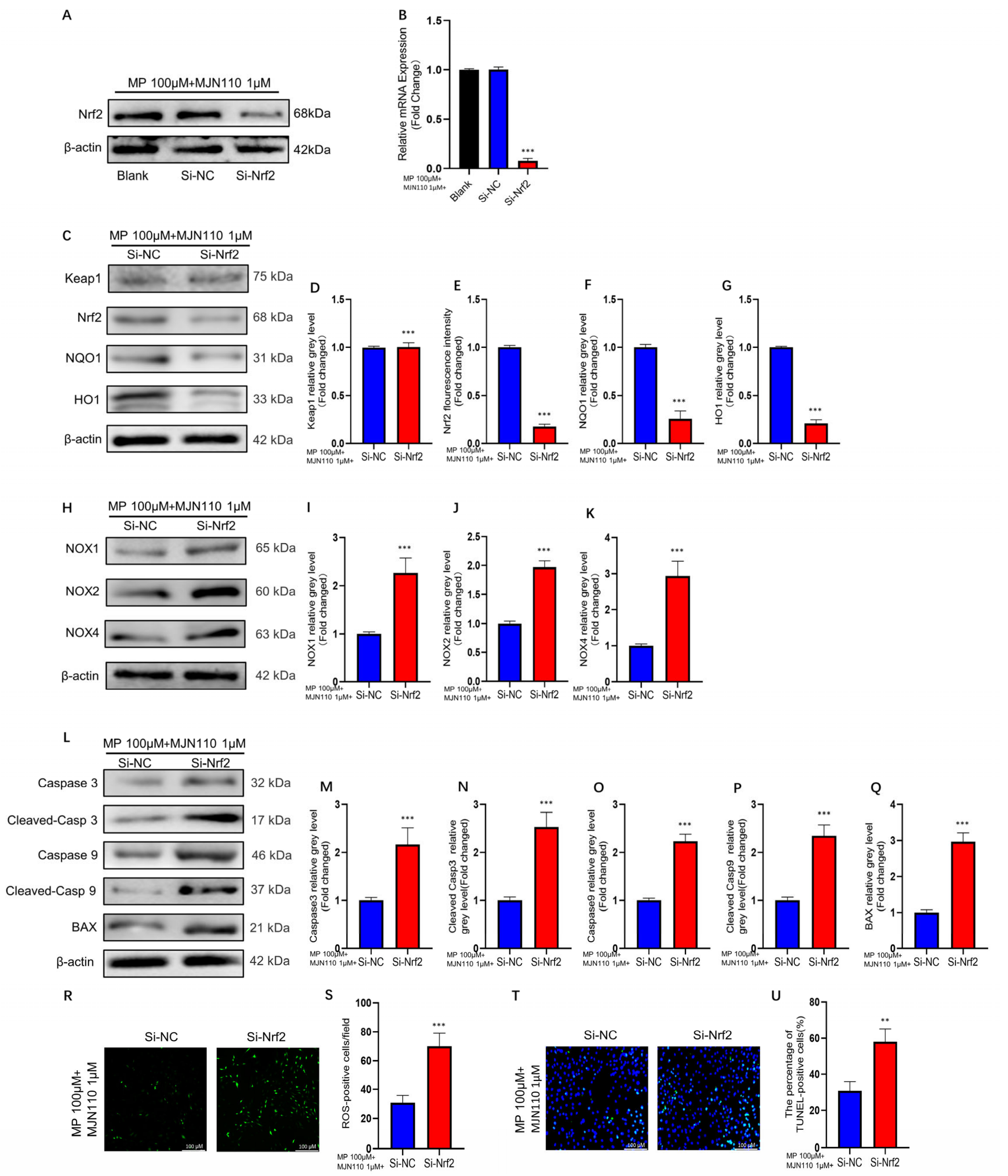

Figure S9: Nrf2 overexpression attenuate GC-induced oxidative stress and apoptosis in BMSCs. (A-B) Overexpression of Nrf2 in BMSCs via Nrf2-expressing plasmid. (C-G) The protein expressions of Keap1, Nrf2, NQO1 and HO1 (NC group versus OV-Nrf2 group). (H-K) The protein expressions of NOX1, NOX2 and NOX4 in both groups. (L-Q) The protein expressions of apoptosis-related proteins in both groups. (R) ROS staining was performed to test the level of oxidative stress in OV-NC group and OV-Nrf2 group. (S) The number of ROS-positive cells per field in both groups. (T) TUNEL staining was performed to test apoptotic rate in OV-NC group and OV-Nrf2 group. (U) The percentage of TUNEL-positive cells in both groups. (n=3, mean ± S.D; *, $P<0.05$; **, $P<0.01$; ***, $P<0.005$ versus OV-NC group). These studies were performed at least 3 biological replicates.

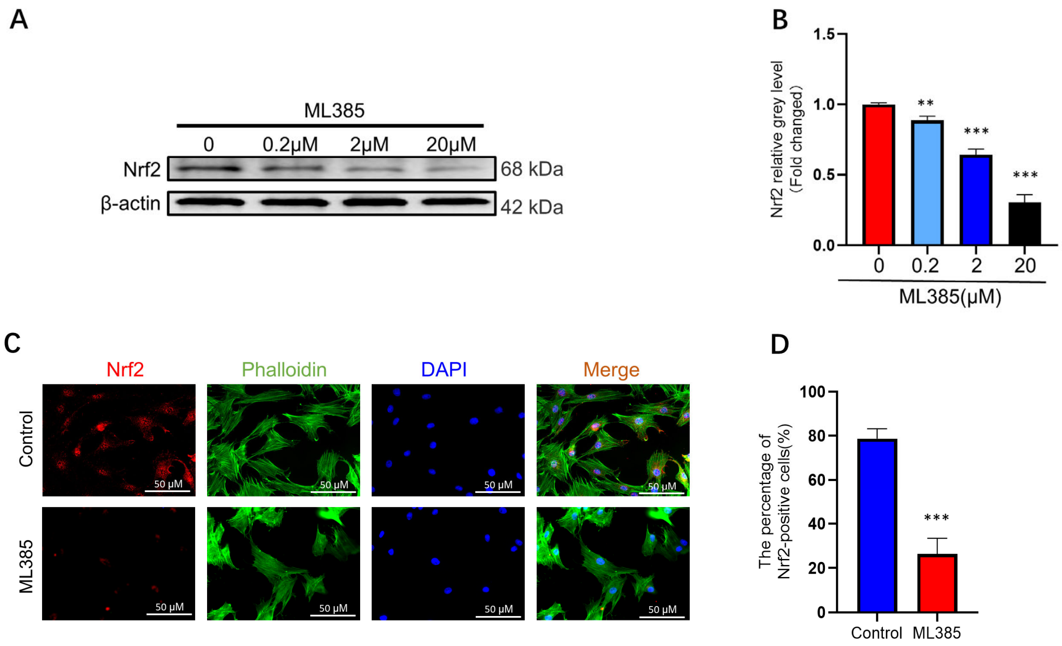

Figure S10: ML385 repressed Nrf2 expression in BMSCs. (A-B) Western blot results for the expressions of Nrf2. BMSCs were incubated in various concentrations of ML385 for 24h. (C) Images of immunofluorescence staining of Nrf2 in control group and ML385 (20μM) group. (D)The percentage of Nrf2-positive cells per field in each group. (n=3, mean ± S.D; *, $P<0.05$; **, $P<0.01$; ***, $P<0.005$ versus control group). This study was performed at least 3 biological replicates.

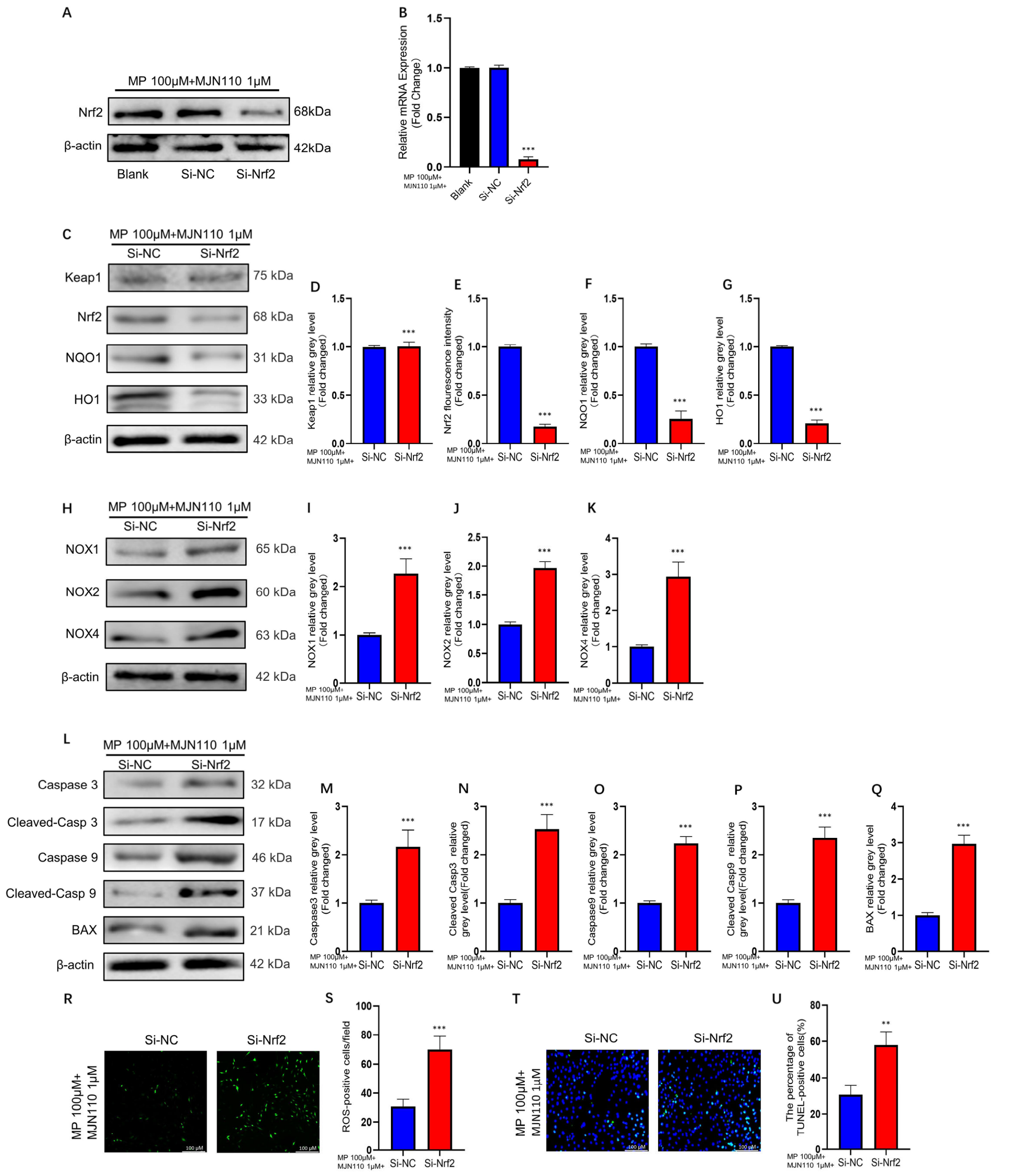

Figure S11: Nrf2-silenced reverses the beneficial effect of MAGL-inhibition on GC-induced oxidative stress and apoptosis in BMSCs. (A-B) Silencing of Nrf2 in BMSCs via siRNA. (C-G) The expressions of Keap1/ Nrf2 signaling pathway proteins in Si-NC group and Si-Nrf2 group. (H-K) The protein expressions of NOX1, NOX2 and NOX4 in both groups. (L-Q) The protein expressions of apoptosis-related proteins in both groups. (R) ROS staining of BMSCs in both groups. (S) The number of ROS-positive cells per field in both groups. (T) TUNEL staining was performed to test apoptotic rate in both groups. (U) The percentage of TUNEL-positive cells in both groups. (n=3, mean ± S.D; *, P<0.05; **, P<0.01; ***, P<0.005 versus Si-NC group). These studies were performed at least 3 biological replicates.

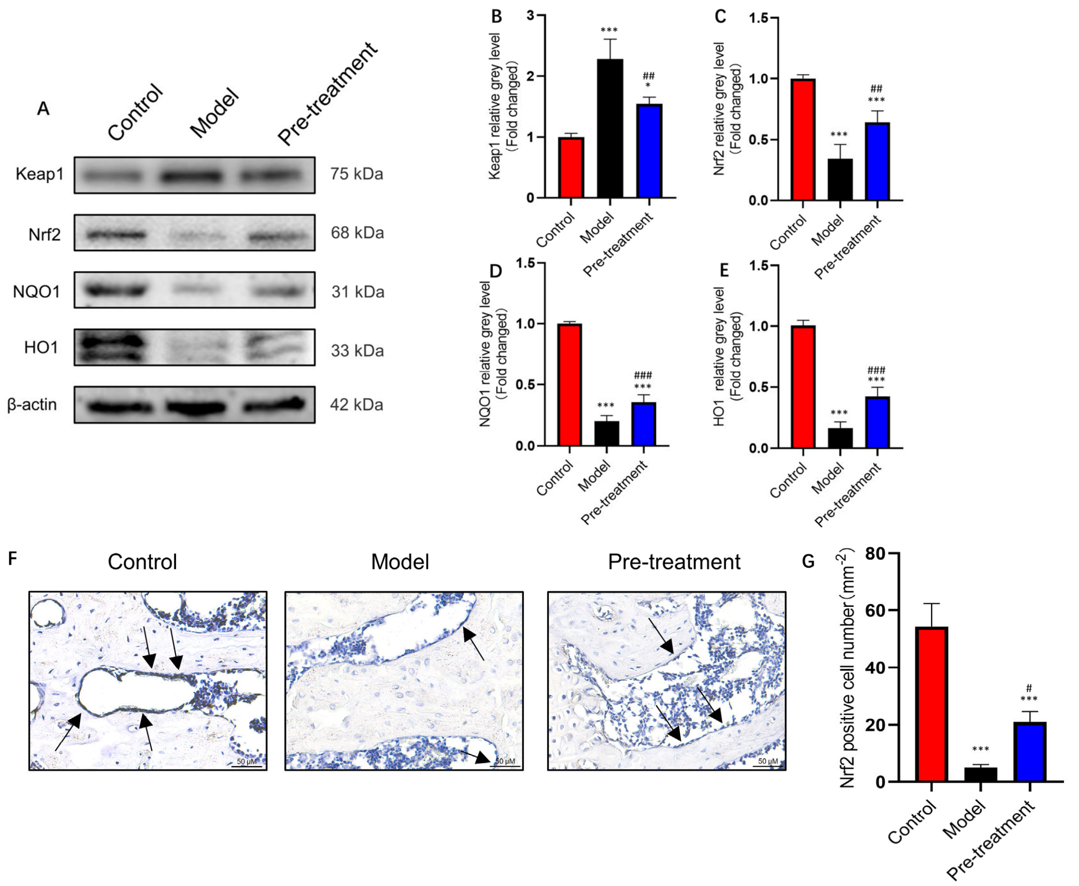

Figure S12: Pre-treatment with MAGL inhibitor activates Keap1/Nrf2 signaling pathway in vivo. (A-E) The protein expression level of Keap1/Nrf2 signaling pathway in bone tissue in each group. (F) IHC staining of Nrf2, the IHC-positive cells were marked with black arrows. (G) Average number of IHC-positive cells per field in each group. (n=5, mean ± S.D; *, P<0.05 versus control group. **, P<0.01; ***, P<0.005; #, P<0.05; ##, P<0.01; ###, P<0.005 versus MP group). All these studies were performed at least 3 biological replicates.

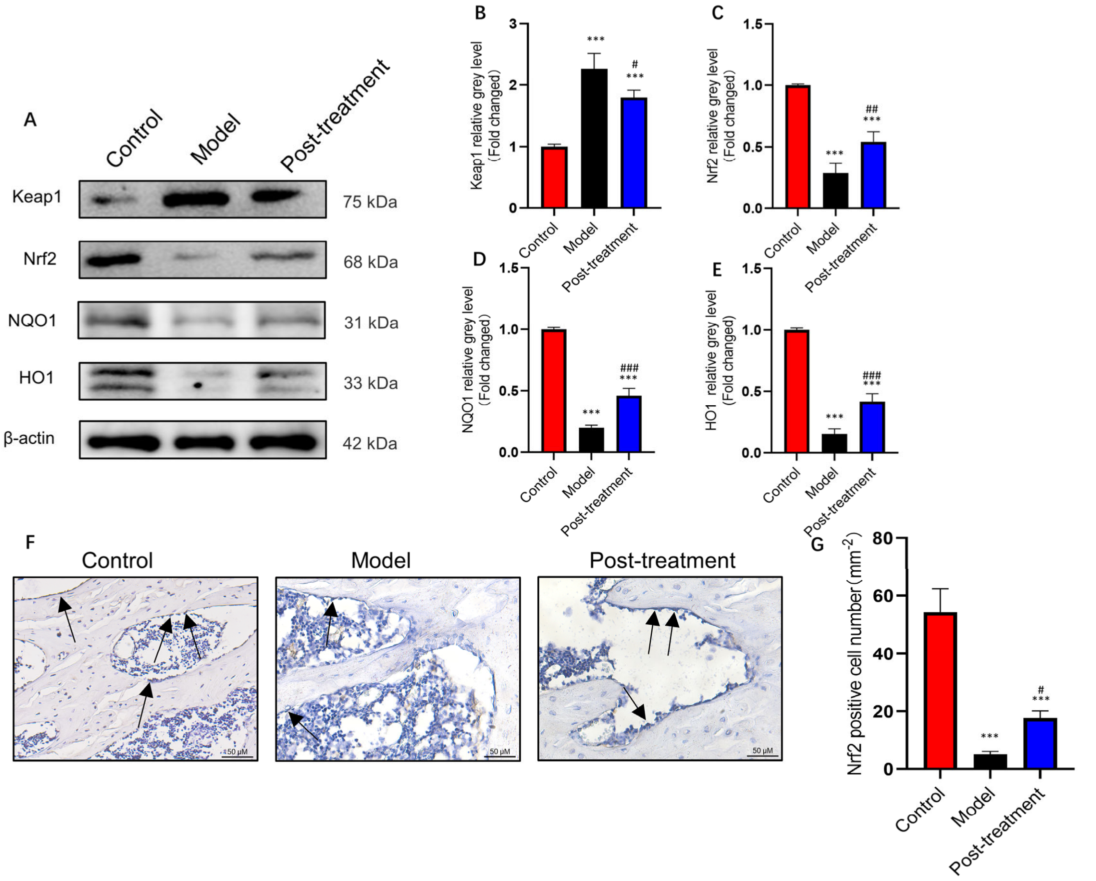

Figure S13: Post-treatment with MAGL inhibitor activates Keap1/Nrf2 signaling pathway in vivo. (A-E) The protein expression level of Keap1/Nrf2 signaling pathway in bone tissue in each group. (F) IHC staining of Nrf2, the IHC-positive cells were marked with black arrows. (G) Average number of IHC-positive cells per field in each group. (n=5, mean ± S.D; *, $P<0.05$; **, $P<0.01$; ***, $P<0.005$ versus control group; #, $P<0.05$; ##, $P<0.01$; ###, $P<0.005$ versus MP group). All these studies were performed at least 3 biological replicates.

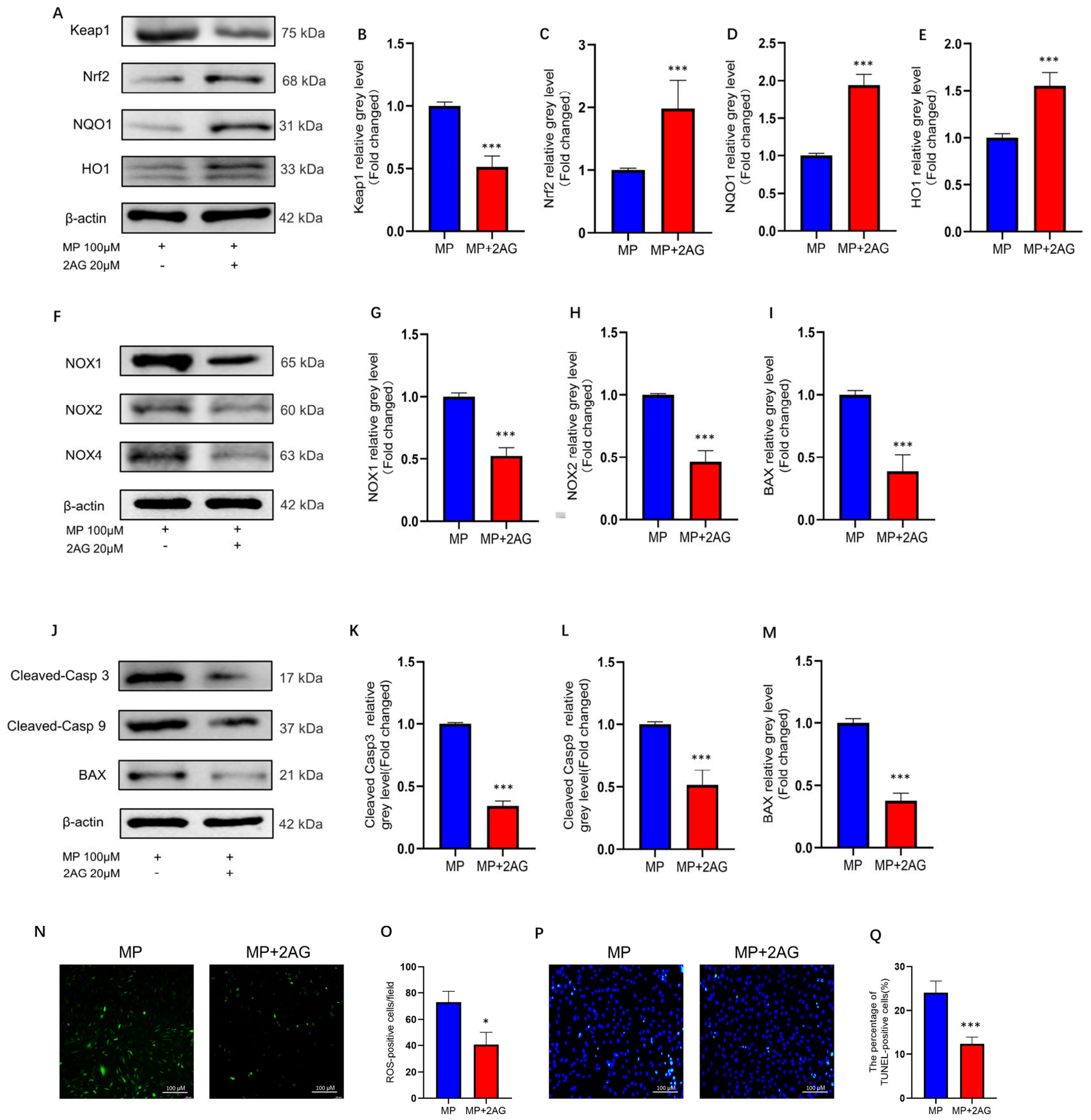

Figure S14: 2AG alleviate GC-induced oxidative stress and apoptosis in BMSCs. (A-M) Western blot results for the expressions of Keap1/Nrf2 pathway proteins, NADPH oxidative isozymes and apoptosis-related proteins. In MP+2AG group, BMSCs were pretreated with 2AG (20μM) for 24 h; MP (100μM) was then added for 24 h or 48 h. (N) ROS staining of BMSCs (MP group versus MP +2AG group). Chronology of drug intervention are the same as that in (F). (O) Average number of ROS positive cells per field in both groups. (P) TUNEL staining was performed to test apoptotic rate in MP group and MP+2AG group. Chronology of drug intervention are the same as that in (J). (Q) Quantitative analysis of the positively TUNEL-stained BMSCs ratio in (P). (n=3, mean ± S.D; **P*<0.05; ***P*<0.01, ****P*<0.005 versus MP group). These studies were performed at least 3 biological replicates.

Table S1. Chemicals and Antibodies

Name	Source	Identifier
Dimethyl sulfoxide (DMSO)	Sigma-Aldrich	CAS#67-68-5
Lipopolysaccharide (LPS)	Sigma-Aldrich	EINECS#297-473-0
Diphenyleneiodonium chloride (DPI)	Sigma-Aldrich	CAS#4673-26-1
VAS2870	MCE	CAS#722456-31-7
Curcumin	Sigma-Aldrich	CAS#458-37-7
ML385	Sigma-Aldrich	CAS#846557-71-9
Methylprednisolone	Pfizer Manufacturing	CAS#82-43-2
MJN110	Cayman Chemicals	CAS#1438416-21-7
2Arachidonoylglycerol (2AG)	MCE	CAS#53847-30-6
NOX1 antibody	Abcam	Ab131088
		Dilution: WB 1:1000; IHC1:1000
NOX2 antibody	Abcam	Ab129068
		Dilution: WB 1:1000
NOX4 antibody	Abcam	Ab109225
		Dilution: WB 1:2000; IHC1:500
Caspase 3 antibody	Abcam	Ab131088
		Dilution: WB 1:500
Cleaved Caspase 3 antibody	Affinity	Ab2846190
		Dilution: WB 1:2000
Caspase 9 antibody	Abcam	Ab2013
		Dilution: WB 1:1000
Cleaved Caspase 9 antibody	HUABIO	ER60008
		Dilution: WB 1:2000
BAX antibody	Abcam	Ab32503
		Dilution: WB 1:5000
MAGL antibody	Abcam	Ab77398
		Dilution: WB 1:1000; IHC1:100; IF 1:50
Keap1 antibody	Abcam	Ab118285
		Dilution: WB 1:1000

Nrf2 antibody	Abcam	Ab89443<br>Dilution: WB 1:500; IF 1:500
NQO1 antibody	Abcam	Ab28947<br>Dilution: WB 1:1000
β-actin antibody	Abcam	Ab6276<br>Dilution: WB 1:5000
Phalloidin-iFluor 488	Abcam	Ab176753<br>Dilution: IF 1:300
Alexa Fluor@647	Abcam	Ab150135 and 150115<br>Dilution: IF 1:100
Goat Anti-Rabbit IgG secondary antibody	Abcam	Ab205718<br>Dilution: WB 1:5000
Goat Anti-Mouse IgG secondary antibody	Abcam	Ab6708<br>Dilution: WB 1:5000
DAPI	Abcam	Ab104139<br>Dilution: IF 1:20
Plasmid pcDNA3.1 (+) (5′-3′) MAGL	GenePharma	CCTGAGGCAAGTTCACCAGGCGAACTCCA CAGAACGTCCCCTACCAGGACCTTCCTCAC
Plasmid pcDNA3.1 (+) (5′-3′) Nrf2	GenePharma	TTTGATTGACATCCTTTGGAGGCAAGACATA GATCTTGGGGTAAGTCGAG AAGTGTTTGA
LV3(H1/GFP&Puro) lentiviral vector sh-MAGL	GenePharma	5′-GCGTGCTGTCTCGGAACAAGT-3′
LV3(H1/GFP&Puro) lentiviral vector sh-NC	GenePharma	5′-TTCTCCGAACGTGTCACGT-3′
RNA oligo si-Nrf2	GenePharma	sense:5′GGGUAAGUCGAGAAGUGUUTT3′;<br>antisense:5′AACACUUCUCGACUUACCCTT3′
RNA oligo si-NC	GenePharma	sense:5′UUCUCCGAACGUGUCACGUTT3′;<br>antisense:5′ACGUGACACGUUCGGAGAATT3′
MAGL primer sequences	Sangon	sense:5′GCTGCAGAAGCAAGAGAACC3′;<br>antisense:5′GGCAGTGAAGACTGAACTTTCA3′
Nrf2 primer sequences	Sangon	sense:5′CCGCAGAGCATTCCCTACCA3′;

			antisense:5′GCTGCAACACATCCCTGACG3′
GAPDH sequences	primer	Sangon	sense:5′GCAAGTTCAACGGCACAG3′; antisense:5′CGCCAGTAGACTCCACGAC3′

IHC, immunohistochemistry; IF, immunofluorescence; WB, Western blot; GAPDH: glyceraldehyde 3-phosphate dehydrogenase.
